# Integrating bulk and single-cell RNA sequencing analysis to reveal characterization of mechanical stimulus-related genes and prognostic signatures in breast cancer

**DOI:** 10.1186/s13058-025-02130-6

**Published:** 2025-11-13

**Authors:** Ze Yang, Haifeng Lou, Yuqiao Huang, Lingyun Guo, Yingfei Huang, Gao Zhu, Jingjia Li, Yindan Lin, Jiang Zhu, Yandi Sun

**Affiliations:** 1 School of Life Science, Zhejiang Chinese Medical University, Hangzhou, 310053 Zhejiang China; 2 Cancer Institute (Key Laboratory of Cancer Prevention and Intervention, China National Ministry of Education), The Second Affiliated Hospital, Zhejiang University School of Medicine, Hangzhou, 310009 China; 3 Liangzhu Laboratory, Zhejiang University Medical Center, Hangzhou, 311121 China; 4 Department of Ultrasound, Women’s Hospital, Zhejiang University School of Medicine, Hangzhou, 310006 China; 5 Zhejiang Provincial Key Laboratory of Precision Diagnosis and Therapy for Major Gynecological Diseases, Women’s Hospital, Zhejiang University School of Medicine, Hangzhou, 310006 China; 6 Department of Otolaryngology, Ningbo No.2 Hospital, Ningbo, Zhejiang China; 7 Zhangjiang Fudan International Innovation Center, Human Phenome Institute, Fudan University, Shanghai, China; 8 Department of Toxicology and Cancer Biology, University of Kentucky College of Medicine, Lexington, KY 40536 USA

**Keywords:** Mechanical stimulus, Breast cancer, Prognostic signature, Tumor immune microenvironment

## Abstract

**Objectives:**

To identify molecular clusters and establish a scoring model based on mechanical stimulus-related genes (MSRGs) for predicting the prognosis of breast cancer patients and understanding the role of mechanical stimuli in the breast tumor microenvironment (TME).

**Methods:**

We utilized bulk and single-cell RNA sequencing analysis to characterize MSRGs associated with breast cancer prognosis. Unsupervised consensus molecular clustering was applied to identify distinct clusters based on overall survival-associated MSRGs from The Cancer Genome Atlas (TCGA) database. The scoring model was constructed by LASSO-Cox method and validated. Additionally, single-cell RNA sequencing analysis, along with in vitro and in vivo experiments, were conducted to further investigate the role of the model in breast cancer.

**Results:**

We identified 23 overall survival-associated MSRGs and established two molecular subgroups with distinct survival outcomes. A prognostic signature incorporating 15 MSRGs was developed and validated, demonstrating its predictive capability for overall survival of breast cancer patients. The nomogram integrating clinical characteristics and the mechanical stimulus-related risk score exhibited promising predictive accuracy. The low-risk group displayed an immune "hot" phenotype with increased immune cell infiltration, while the high-risk group exhibited resistance to conventional chemotherapy but potential sensitivity to Sepantronium bromide. By using the SCISSOR algorithm, we provide evidence at single-cell resolution for the impact of mechanical stimulation on tumor immune microenvironment. The in vivo and in vitro assays demonstrated that knockdown of TEX19 significantly suppressed breast tumor proliferation.

**Conclusion:**

We developed a pioneering prognostic signature incorporating MSRGs in breast cancer, with a particular focus on mechanical stimuli may influence breast cancer prognosis by remodeling the immune microenvironment. The findings highlighted the importance of personalized treatment strategies and provide new insights into the role of mechanical forces in breast tumor biology.

**Supplementary Information:**

The online version contains supplementary material available at 10.1186/s13058-025-02130-6.

## Introduction

Breast cancer is a prevalent type of cancer. According to the data from the GLOBOCAN, breast cancer is the most commonly diagnosed cancer and the leading cause of cancer-related deaths in women [1]. Therapeutic strategies for breast cancer currently encompass local modalities (surgery and radiation) and systemic approaches (chemotherapy, endocrine therapy, and targeted therapy), with treatment selection guided by disease stage and molecular pathogenesis [2, 3]. Despite significant advancements in treatment strategies for breast cancer over the past decades, the disease continues to exhibit a global mortality-to-incidence ratio of approximately 15% [4]. Future research should focus on not only the development of innovative pharmaceutical agents but also on individualizing therapy to accommodate the distinct features of each patient [5]. Hence, the identification of specific biomarkers at an individual level and the exploration of novel therapeutic targets are imperative for advancing treatment strategies and improving clinical outcomes for breast cancer patients.

Solid tumors and tumor microenvironment (TME) contains not only tumor cells but also various stromal components such as the extracellular matrix (ECM), basement membrane, immune cells, fibroblasts, and vasculature, which are involved in tumor progression through alteration of their physical configurations and functionalities [6]. For instance, the mean elasticity values for invasive breast cancers were found to be approximately 140 kPa, which is nearly five times higher than the average of 28 kPa observed in benign lesions [7]. The increased stiffness of tumor tissue is primarily attributed to the excessive deposition and enhanced crosslinking of the ECM [8]. Mechanoimmunology is an emerging field that has recently garnered widespread attention. Breast cancer tissue samples with a high collagen density in the ECM surrounding the tumor exhibit reduced infiltration of T cells and impaired capacity of effector T cells to eliminate cancer cells [9, 10]. Mechanical stimulation can affect cell estimation and signal transduction through mechanosensors and mechanosensitive biomolecules including integrin, cadherin, GPCR, Piezos, and YAP/TAZ [6, 11]. For example, Piezo1 channels induce calcium influx and downstream Src signaling, mediating enhanced invasion of breast cancer cells [12]. The EPHA2/LYN/TWIST1 mechanotransduction pathway responds to mechanical signals from the tumor microenvironment, promoting breast cancer epithelial-to-mesenchymal transition and invasion [13]. However, the specific role of mechanical stimulation in breast cancer and its potential impact on disease development or progression requires further exploration. Meanwhile, the response of tumor-infiltrating immune cells to the diverse mechanical stimulus within tumors has been barely examined [14]. Therefore, a comprehensive analysis of genetic alterations in genes related to mechanical stimulation could uncover molecular targets for future breast cancer therapeutic.

In this study, we aimed to identify distinct molecular clusters and establish a scoring model based on mechanical stimulus-related genes (MSRGs) to understand their roles in the breast tumor immune microenvironment (TIME) and predict the prognosis of breast cancer patients. Firstly, unsupervised consensus molecular clustering was used to obtain distinct clusters related to mechanical stimulus based on the overall survival-associated MSRGs in The Cancer Genome Atlas (TCGA) database. Then, a 15-genes scoring model was built using the LASSO-Cox method and subsequently validated. In particular, we performed a combined analysis of single-cell sequencing data and bulk RNA sequencing to reveal the impact of the MSRG signature on the TIME. Lastly, in vitro and in vivo experiments were conducted to elucidate the role of the key model gene TEX19 in the development of breast cancer. To the best of our knowledge, this represents the first prognostic signature in breast cancer that integrates MSRGs, indicating that mechanical stimuli influence breast cancer prognosis by remodeling the immune microenvironment. This offers a novel approach to clinical management and provides valuable insights into the treatment of breast cancer.

## Methods

### Dataset gathering and collection of mechanical stimulus-related genes

Regarding the training set, the mRNA expression data, indexed clinical data pertaining to breast cancer (BRCA) were obtained from The Cancer Genome Atlas (TCGA) database using the “SummaryExperiment” and “TCGAbiolinks” [15]. Both raw read counts and TPM (Transcripts Per Million) values were retrieved; raw counts were used for differential expression analyses, whereas TPM values were log_2_-transformed for visualization, clustering, and immune infiltration analyses. A total of 1,058 primary solid tumor samples with clinical and overall survival (OS) data were included in the analysis.

For the external validation set, we obtained normalized and log_2_-transformed microarray-based gene expression profile data and clinical data of 1,980 breast tumor samples from the Molecular Taxonomy of Breast Cancer International Consortium (METABRIC) database [16], downloaded via the cBioPortal for Cancer Genomics (https://www.cbioportal.org/study/summary?id=brca_metabric). In addition, two independent GEO cohorts, GSE20685 [17] and GSE96058 [18], were incorporated as external validation sets; both datasets provided pre-normalized expression matrices and corresponding clinical survival information, which were primarily used to further validate the robustness of our risk score model.

Given that all datasets (TCGA, METABRIC, GSE96058, and GSE20685) were publicly available and accessible to all without restrictions, ethical considerations corresponding to data usage and privacy were not applicable in this study. Baseline clinicopathological characteristics of TCGA and METABRIC were summarized in Supplementary Table 1.

We acquired a set of 213 genes linked to the response to mechanical stimulus from the Molecular Signatures Database v2023.2.Hs (MSigDB, http://www.gseamsigdb.org/gsea/index.jsp) (GO:0009612). Following the intersection with the complete set of genes included in TCGA data set, a total of 212 MSRGs were subsequently retrieved for further analysis (Supplementary Table 2).

### Mutation landscape and correlation of overall survival-associated mechanical stimulus-related genes

In order to elucidate the inherent prognostic significance of the 212 MSRGs in breast cancer, the overall survival (OS)-associated MSRGs were identified through univariate Cox hazard regression analysis with a significance threshold of *p* < 0.05 in the TCGA-BRCA dataset (n = 1,058). The mutation data, specifically categorized as Simple Nucleotide Variation (SNV) and Masked Somatic Mutation with open access, were retrieved from the TCGA-BRCA collection using the “TCGAbiolinks” package [15]. Subsequently, the SNV data of the OS-associated MSRGs were analyzed using the R package “maftools” [19]. Additionally, the gene copy number variation (CNV) of breast cancer was acquired from Xena (https://xenabrowser.net/datapages/) and the CNV frequency of the OS-associated MSRGs was analyzed. We utilized the “RCircos” package [20] to visualize the genomic locations of these OS-associated MSRGs on chromosomes and a correlation matrix plot was generated to illustrate the correlation patterns among these genes.

### Unsupervised consensus molecular clustering

We employed the “ConsensusClusterPlus” package [21] to conduct consensus clustering and identify distinct clusters related to mechanical stimulus based on the expression levels of the OS-associated MSRGs. 90% of the samples were randomly resampled 1000 times to ensure robust clustering results. The consensus clustering parameter “maxK” was set to “8”, “clusterAlg” was designated as “pam”, and “distance” was specified as “pearson”. The optimal number of clusters was determined based on the cumulative distribution function (CDF) plot. The stability of the cluster groups was validated using the principal component analysis (PCA) algorithm. Furthermore, Kaplan–Meier survival analysis was employed to assess the overall survival of the different clusters with the “survival” [22], “survminer” [23] and “TSHRC” [24] packages.

### Construction and validation mechanical stimulus-related prognostic signature for breast cancer cases

To identify differentially expressed genes (DEGs) among 2 clusters, we employed the "limma" package [25]. DEGs were screened based on the following criteria: an adjusted p-value < 0.05 and a |log_2_ fold change (FC)|> 1.5. Subsequently, Univariate Cox regression was used to assess the impact of these DEGs on the overall survival status of breast cancer cases. To refine the selection of OS-related DEGs, we employed the Least Absolute Shrinkage and Selection Operator (LASSO) Cox regression analysis. The “lambda.min” value, determined using the “glmnet” package [26], was utilized to identify the most suitable set of genes for the signature construction. Ultimately, the risk score for each patient was computed using the following formula: Risk score = (expression of Gene 1) * β1 + (expression of Gene 2) * β2 + … + (expression of Gene n) * βn, where β represents the regression coefficient associated with each gene in the signature.

The breast cancer cases in each cohort were stratified into high- and low-risk groups using the median risk score as the cutoff. We utilized the “ggalluvial” package [27] to create an alluvial diagram illustrating the relationships among gene clusters, risk scores, and survival events. To assess the feasibility of the prognostic signature, Kaplan–Meier analysis of overall survival was conducted separately for the high- and low-risk groups in the training and validation datasets. We further compared the differences in clinical features between the two risk groups and visualized these distinctions with the heatmap.

### Development and assessment of the clinical nomogram

Furthermore, we performed both univariate and multivariate Cox regression analyses to determine whether the risk score served as an independent prognostic predictor for breast cancer. Subsequently, leveraging the results obtained, we developed a clinicopathologic nomogram related to MS that integrated the risk score along with two other clinical characteristics, namely age and AJCC stage, in the training set. This nomogram was constructed using the “rms” package. We employed a calibration plot, utilizing the “boot” method with 1000 iterations, to compare predicted and observed overall survival and assess the efficacy of the prognostic nomogram. We also utilized the “time-ROC” package [28] to conduct 1-year, 3-year, and 5-year ROC curve analysis, evaluating the performance of the nomogram.

### Function enrichment analysis of two risk groups

With “limma” package [25], the DEGs between the low- and high-risk groups were identified based on the specified criteria (|log2FC|> 1 and the adjusted p-value < 0.05). Then, pathway and functional annotations were conducted through Gene Ontology (GO) and Kyoto Encyclopedia of Genes and Genomes (KEGG) enrichment analyses using the “clusterProfiler” package [29].

### Assessment of the tumor-immune microenvironment landscape

To dissect the tumor-immune microenvironment landscape between the different groups, the CIBERSORT deconvolution method [30] was used to estimate the abundance of 22 tumor immune-infiltrating cell types under 1000 permutations within the gene expression matrix of the training set. Furthermore, we employed the ESTIMATE algorithm [31] to calculate the estimate scores, immune scores, and stromal scores to predict tumor purity and analyze the tumor microenvironment. Single-sample gene set enrichment analysis (ssGSEA) [32] was also conducted to quantify and compare the enrichment scores of 28 immunocyte subpopulations within the samples.

### Drug sensitivity prediction

We used the Genomic of Drug Sensitivity in Cancer (GDSC) database and the “oncoPredict” package [33] to predict the half-maximal inhibitory concentration (IC50) of potentially effective drugs for breast cancer [34]. We conducted an analysis of the correlation between the IC50 of drugs, risk score, and the model genes. The IC50 of drugs between two risk groups was compared and displayed via the “ggstatsplot” package [35].

### Single-cell RNA sequencing data analysis

We downloaded data for human breast cancers from Kuett L et al., via the Broad Institute Single Cell portal at https://singlecell.broadinstitute.org/single_cell/study/SCP1039. Cells that expressed more than 200 genes and had a mitochondrial percentage of less than 20% were used for downstream analysis. Data normalization, dimensionality reduction and clustering were performed by the Seurat v.4.0.0 package in R software with default parameters [36], and the batch effect was removed using harmony v.1.2.3 package [37]. We retained the high-level annotation from the original authors as cell type annotation. To quantify the similarity between single-cell and bulk data, we employed the “SCISSOR” R package developed by Sun et al., which calculates the Pearson correlation for each cell-bulk pair [38]. The correlation matrix was then optimized based on sample phenotypes, enabling the confident selection of phenotype-relevant cells. The communication network between cells in the tumor microenvironment (TME) was analyzed and visualized using the “CellChat” package [39]. Metabolic scores were calculated with the “scMetabolism” R package [40]. All code was based on the package’s existing scripts, with default parameters used.

### Cell lines and culture

Human breast cancer cell lines MCF-7, MDA-MB-231, BT549, and human mammary epithelial cells MCF10A were purchased from Cell Bank of Type Culture Collection of the Chinese Academy of Sciences, Shanghai, China, which was proven to be free of mycoplasma contamination. MCF-7 and BT549 were cultured in Dulbecco Minimal Essential Medium, MDA-MB-231 was cultured in L-15 medium , and MCF10A was cultured in MEBM

basal medium with 100ng/mL cholera toxin, which were supplemented with 1% penicillin/streptomycin sulfate and 10% fetal bovine serum in the 37 °C cell incubator with 5% CO_2_ atmosphere.

### Generation of TEX19 knockout stable cells

Using PLKO.1 hygromycin plasmid construct, we established MCF-7 stable cell lines with endogenous TEX19 knockout. To generate TEX19 KO MCF-7, two target regions of TEX19 were targeted, the targeted sequences were listed as follows:

TEX19-1 (F): AGGATTCACCATAGTCTCTTA.

TEX19-2 (F): TTCAACATGGAGATCAGCTAA.

### Western blotting

The cells were lysed using HEPES lysis buffer and boiled at 95℃ for 10 min, then the lysates were loaded, electrophoresed for one hour and transferred to the PVDF membrane. The membrane was blocked with 5% non-fat milk for 1 h at room temperature, incubated with antibody overnight at 4℃ and the second antibody at room temperature for another hour. Eventually the membrane was imaged using ECL solution.

### CCK-8 assay

1,000 cells were seeded in the 96-well plate and examined for 24, 48, 72, 96 and 120 h. 10µL CCK-8 work solution was added in every well and incubated for 30 min, and the absorbance was analyzed at OD 450 nm.

### Colony formation assay

One thousand cells (MCF-7 shRandom and shTEX-19) were seeded in the 6-well plate in triplicates and incubated for 8 days with DMEM medium, Viable colonies were stained with crystal violet and counted through Image J software.

### Flow cytometry assay

Cells were fixed with 70% cold-ethanol at 4℃ for 30 min, washed twice and stained by the mixture containing RNase (100 µg/mL) and PI (50 µg/mL) for 30 min. The fluorescence intensity was detected through flow cytometry and analyzed by Flowjo software.

### Mice

All animal procedures were conducted with the approval of the Animal Research Ethics Committee at Zhejiang University. BALB/c nude mice (4 weeks old) were purchased from Slaccas animal Company. All the mice were housed in Laboratory Animal Center of Zhejiang University with stable temperature (23 ± 2°C) and light/dark at the interval of 12 h.

### Xenograft mouse model

Mice were injected subcutaneously with 5 × 10^6^ stable cells (n = 6 for each group). At day 10, tumor growth was measured every 3 days, and tumor volume was calculated as the formula of 1/2 × major diameter × minor diameter^2^. When the major diameter reached the maximally permitted condition, mice were sacrificed. After dissection, the weights of tumors were measured and recorded.

### Statistical analysis

All bioinformatics statistical analyses were conducted via R software version 4.3.3. The Wilcoxon test, also known as the Mann–Whitney U test, was used to evaluate differences between two groups. The Kruskal–Wallis test was used to assess differences among three or more groups and the Holm-Bonferroni test is applied for subsequent comparisons between two groups within these groups. The correlations between the IC50 of drugs, risk score, and the model genes were calculated with spearman rank correlation. *p* value for multiple correlations was adjusted by “fdr” method. If the survival curves did not intersect, the Kaplan–Meier curve and log-rank test were used to assess survival differences between groups. If the survival curves crossed, a two-stage analysis was applied [41]. For statistics analyses of in vitro and vivo experiments, the data were analyzed with student’s t-test, or two-way analysis of variance (ANOVA). For animal models, we underwent a randomization process in mice within the same groups. A statistical significance level of *p* < 0.05 was considered to determine statistical significance in the analysis.

## Result

### Workflow of this study

**Fig. 1:**
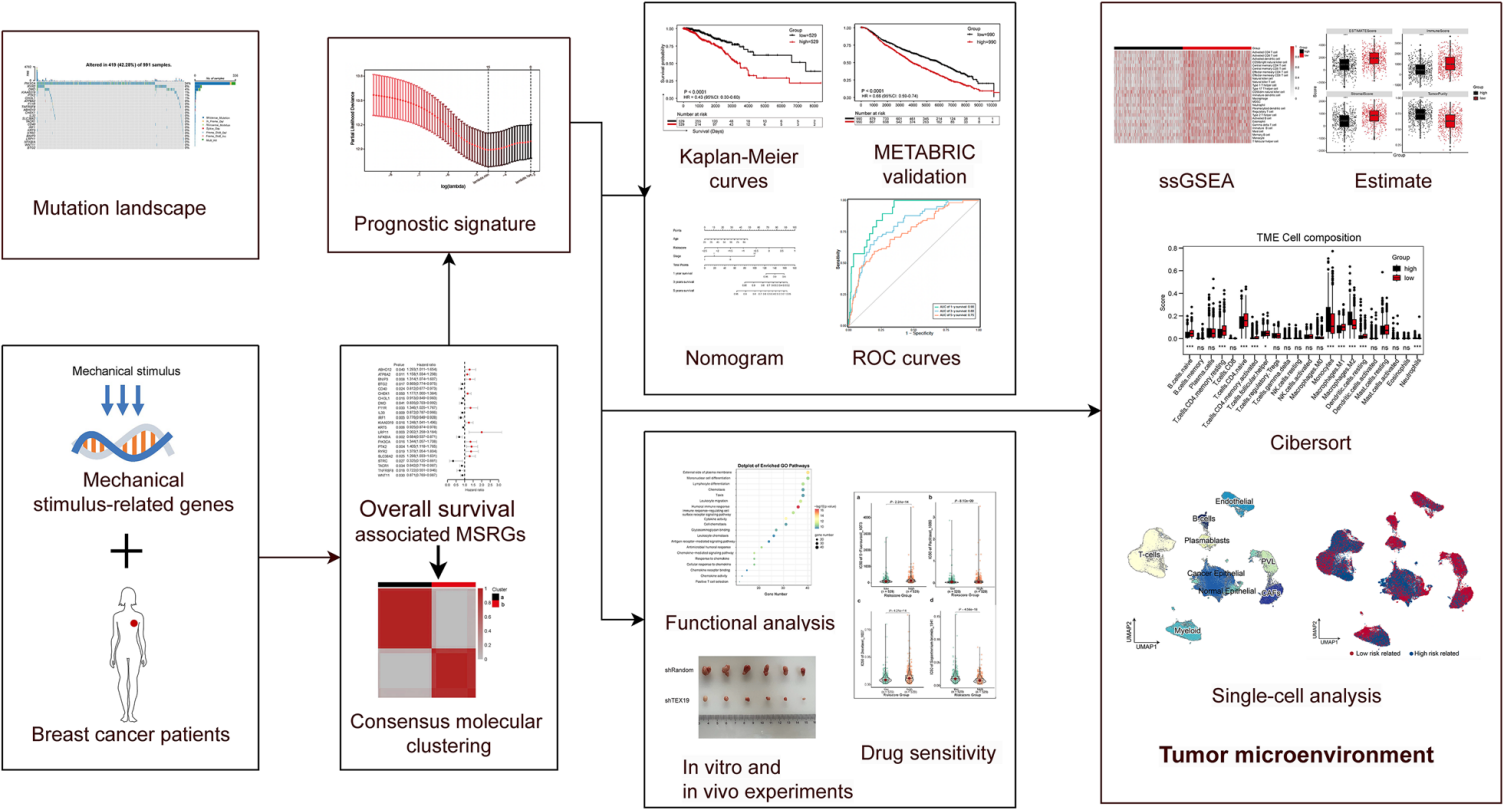
Flowchart outlining the comprehensive analysis of mechanical stimulus patterns in breast cancer patients

The overall workflow of our study is depicted in Fig. 1.

### Genetic variation landscape of OS-associated MSRGs in breast cancer

**Fig. 2:**
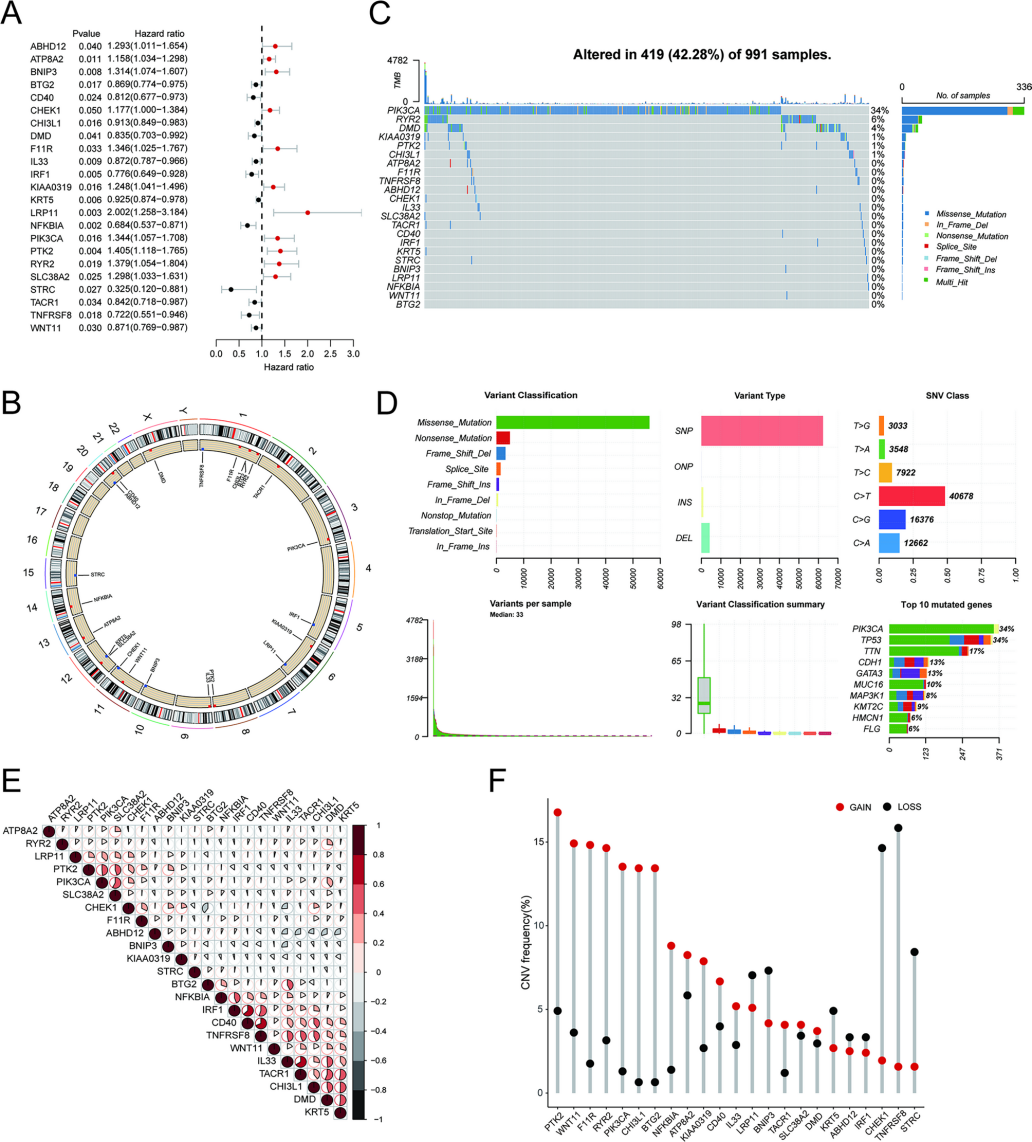
Variant landscape of mechanical stimulus-related genes (MSRGs) associated with overall survival (OS) in breast cancer patients. **A** OS-associated MSRGs identified through univariate Cox regression analysis. **B** Chromosomal locations of OS-associated MSRGs. **C**-**D** Oncoplot depicting the frequency of OS-associated MSRG alterations in the TCGA cohort. **E** Correlation network of OS-associated MSRGs. **F** Copy number variation (CNV) frequency of OS-associated MSRGs in the TCGA cohort

Following univariate Cox regression analysis, 23 OS-associated MSRGs in BRCA were selected for further analyses (Fig. 2A). A Circos plot was used to illustrate the chromosomal locations of 23 OS-associated MSRGs (Fig. 2B). In the somatic mutation analysis, 419 out of the 991 samples (42.28%) exhibited mutations. Among these mutations, missense mutations were the most prevalent, with PIK3CA showing the highest mutation rate (Fig. 2C,D ). The correlation network plot demonstrated the expression correlations between the 23 genes (Fig. 2E). Furthermore, an analysis of the frequency of CNV in the OS-associated MSRGs revealed that both amplification and loss of copy number were widespread (Fig. 2F).

### Consensus molecular clustering analysis

**Fig. 3:**
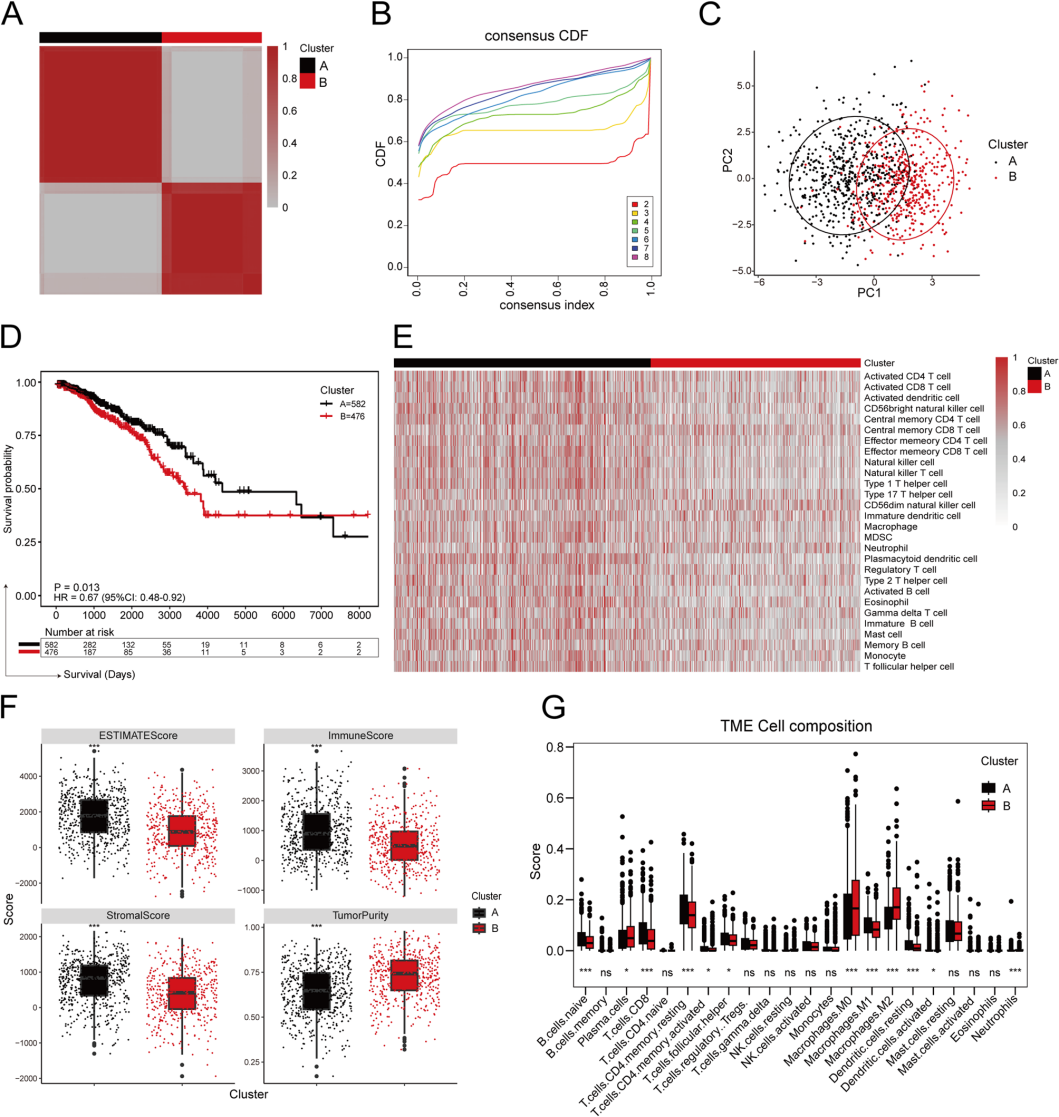
Unsupervised clustering of mechanical stimulus-related genes (MSRGs). **A** Heatmap of consensus matrices for k = 2. **B** Cumulative distribution function (CDF) plot of consensus clustering. **C** Principal component analysis (PCA) of transcriptome profiles across the two clusters. **D** The Kaplan–Meier curves of breast cancer patients from the TCGA cohort based on the two clusters. Statistics, Two-stage analysis. **E** Heatmap displaying ssGSEA results of the two clusters. (**F**) Differences in ESTIMATE score, stromal score, immune score, and tumor purity between the two clusters. Statistics, Wilcoxon test. *p < 0.05; **p < 0.01; ***p < 0.001. **G** Boxplots illustrating differences in the infiltration levels of 22 immune cell types between the two clusters. Statistics, Wilcoxon test. *p < 0.05; **p < 0.01; ***p < 0.001

Consensus clustering was conducted to identify molecular clusters based on the expression levels of OS-associated MSRGs. K = 2 was determined as the stable aggregation based on the results depicted in Fig. 3A-B. Consequently, all breast cancer samples were categorized into two molecular subgroups: Cluster A (n = 582) and Cluster B (n = 476). PCA demonstrated a distinct separation between the two clusters (Fig. 3C). Compared with Cluster B, Cluster A exhibited a significant prolongation of overall survival (*P* = 0.013, HR = 0.67, 95% CI: 0.48–0.92; Fig. 3D). As showed in the ssGSEA results (Fig. 3E), it was evident that patients in Cluster A, who had a better prognosis, displayed an immune “hot” phenotype in their tumor microenvironment. The ESTIMATE analysis indicated that the patients in Cluster A exhibited elevated estimate scores, immune scores, and stromal scores, alongside reduced tumor purity compared to patients in Cluster B (Fig. 3F). The CIBERSORT algorithm unveiled that the TME of patients in Cluster A showed significant enrichment of naive B cells, CD8^+^ T cells, CD4^+^ memory T cells, macrophages M1. Conversely, patients in Cluster B exhibited a notable increase in M0 and M2 macrophages within the TME (Fig. 3G).

### Construction and validation mechanical stimulus-related prognostic signature

**Fig. 4:**
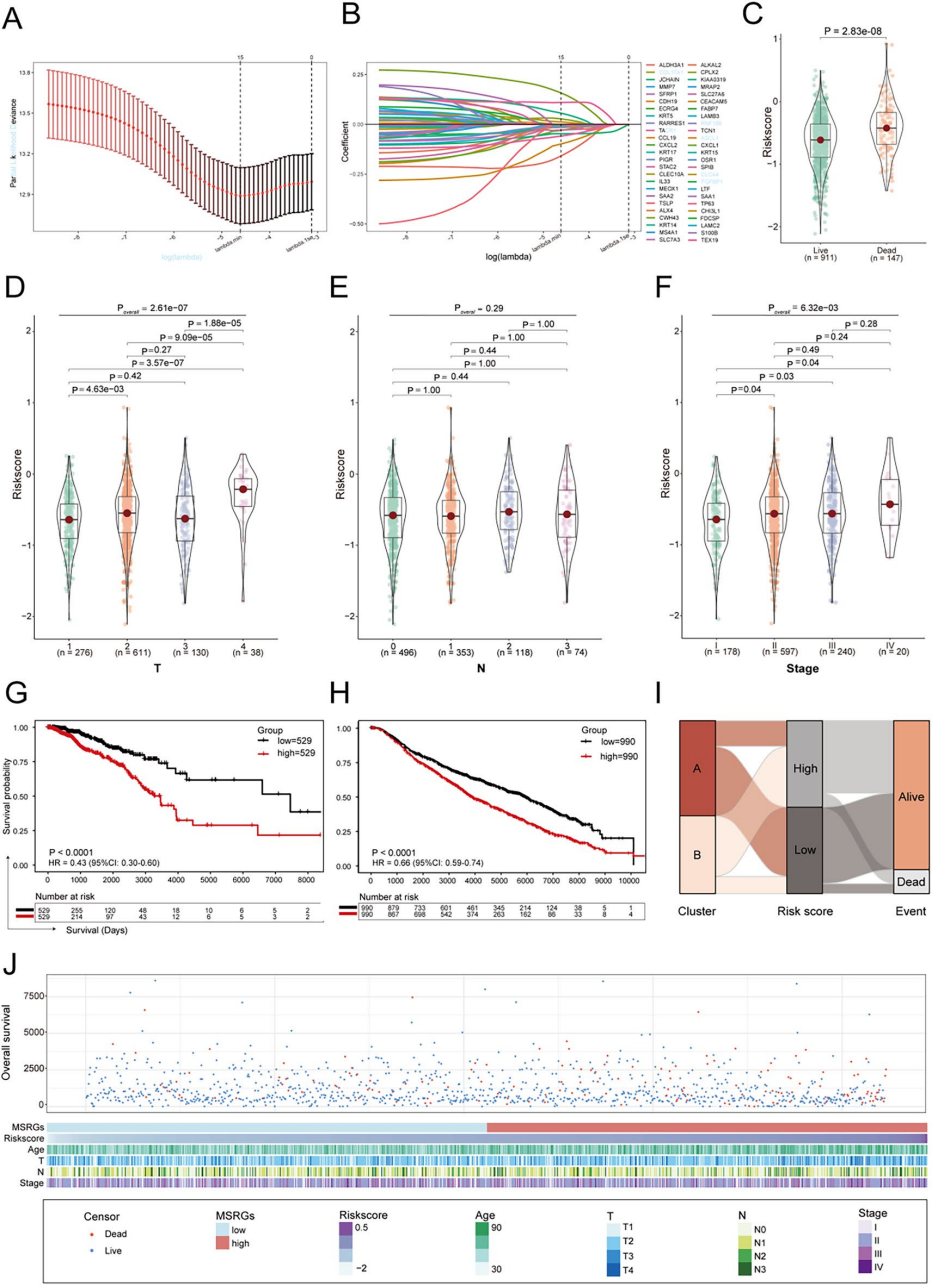
Construction of a prognostic gene signature for breast cancer patients. **A**-**B** Lasso regression analysis of OS-associated DEGs. **C**-**F** Violin plots showing the relationship between risk score and survival status, tumor stage (T), nodal status (N), and clinical stage. Statistics, Wilcoxon test for Fig C, Holm-Bonferroni method for Fig D-F to compare the two groups, and Kruskal–Wallis test for Fig D-F to compare all groups. **G** The Kaplan–Meier curves of low- and high-risk score groups based on breast cancer patients from the TCGA cohort. Statistics, log-rank test. **H** The Kaplan–Meier curves of low- and high-risk score groups based on breast cancer patients from the METABRIC cohort. Statistics, Two-stage analysis. **I** Alluvial diagram illustrating the interrelationship between molecular clusters, risk groups, and survival status of breast cancer patients. **J** Relationship between overall survival, risk scores, and clinicopathological features in the TCGA cohort

In the training set, 260 DEGs (adjusted *p* < 0.05, and |log2FC|> 1.5) were identified, with 242 downregulated genes and 18 upregulated genes in Cluster B versus Cluster A, as illustrated in a volcano plot (Supplementary Fig. 1, Supplementary Table 3). We then identified 50 OS-associated DEGs using Univariate Cox regression with a significance criterion of *p* < 0.05 for further analysis (Supplementary Table 4). Through the application of LASSO Cox regression analysis in breast cancer cases, 15 pivotal genes were kept to establish the prognostic signature (ALDH3A1, CCL19, CDH19, CEACAM5, CPLX2, CWH43, CXCL1, FABP7, JCHAIN, KIAA0319, KRT15, PIGR, SPIB, TCN1, TEX19) and highlighted on the volcano plot (Fig. 4A-B, Supplementary Fig. 1). Based on the prognostic signature, the mechanical stimulus index (MSI) of each patient was calculated as follows: Risk score =—(expression of ALDH3A1) * 0.158859715—(expression of CCL19) * 0.015415473—(expression of CDH19) * 0.098733091 + (expression of CEACAM5) * 0.011877118 + (expression of CPLX2) * 0.027222987 + (expression of CWH43) * 0.157808964—(expression of CXCL1) * 0.030283461—(expression of FABP7) * 0.003265046—(expression of JCHAIN) * 0.047094207 + (expression of KIAA0319) * 0.056197265—(expression of KRT15) * 0.005340380—(expression of PIGR) * 0.012580670—(expression of SPIB) * 0.028761345—(expression of TCN1) * 0.059128906 + (expression of TEX19) * 0.109843007. We further examined gene expression in TCGA and found that several signature genes (TCN1, PIGR, KRT15, JCHAIN, FABP7, CXCL1, CDH19, ALDH3A1) were downregulated in tumors, while others (TEX19, SPIB, KIAA0319, CEACAM5) were upregulated (Supplementary Fig. 2). These patterns were largely consistent with the direction of their regression coefficients, underscoring the robustness of the 15-gene signature. The breast cancer cases were stratified into high- and low-risk groups separately in both the training and validation sets based on the risk score calculated using the aforementioned formula. Risk score was significantly associated with various clinical features, including survival status (live or dead), tumor stage (T1–T4), and clinical stage (I–IV), except for lymph node involvement (N0–N3) (Fig. 4C-F). The Kaplan–Meier curves for the overall survival of breast cancer patients demonstrated that individuals with a low-risk score exhibited significantly longer overall survival across both cohorts (TCGA: *P* < 0.0001, HR = 0.43, 95% CI: 0.30–0.60; METABRIC: *P* < 0.0001, HR = 0.66, 95% CI: 0.59–0.74; Fig. 4G-H). Furthermore, in the GSE96058 and GSE20685 cohorts, the low-risk group also demonstrated more favorable outcomes, supporting the generalizability of our prognostic signature across independent datasets (GSE96058: *P* = 0.0036, HR = 0.73, 95% CI: 0.59–0.90; GSE20685: *P* = 0.0030, HR = 0.51, 95% CI: 0.32–0.80; Supplementary Fig. 3). To further analyze the relationship between the prognostic signature and clinical characteristics, we performed survival analyses in the TCGA cohort stratified by tumor stage (T1–T4), lymph node involvement (N0–N3) and clinical stage (I–IV), using the median risk score as the cutoff. The results showed that patients in the low-risk group had significantly better survival in most of breast cancer progression (T2, T3, N0, N2, and Stage II、III、IV). In other subgroups, the low-risk group consistently showed a trend toward better outcomes, although the differences did not reach statistical significance, likely due to limited sample size (Supplementary Fig. 4). We utilized a Sankey diagram to compare the risk scores of the two clusters of patients and found that the high-risk group predominantly comprised more than half of the patients in Cluster B, with only a few patients in Cluster A. Meanwhile, an analysis of the grouping of risk score versus patient survival event revealed that a higher number of patients in the high-risk score group experienced a fatal outcome. Figure 4J illustrated the relationship between risk scores, overall survival status, and clinicopathologic features in the TCGA cohort.

### Development and assessment of the prognostic nomogram

**Fig. 5:**
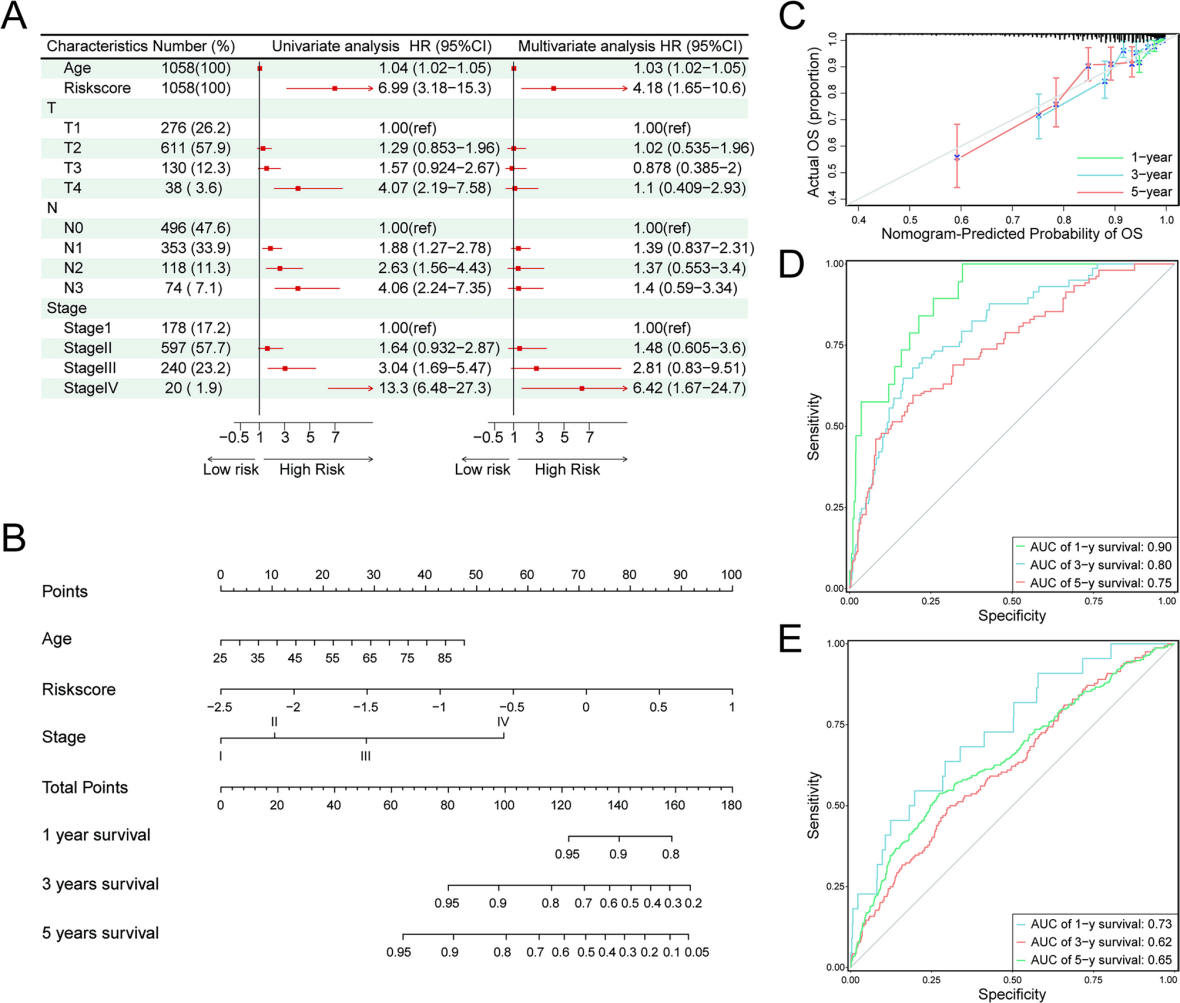
Establishment and evaluation of the nomogram survival model. **A** Univariate and multivariate analysis of clinicopathological characteristics and risk score in the TCGA cohort. **B** Construction of a nomogram to predict the prognosis of breast cancer patients. **C** Calibration plots showing the predicted probabilities of 1-, 3-, and 5-year overall survival in the TCGA cohort. **D** Receiver operating characteristic (ROC) analysis of the nomogram in the TCGA cohort. **E** ROC analysis of the nomogram in the METABRIC cohort

Univariate and multivariate Cox regression analyses were conducted to evaluate the prognostic significance of the risk score variable, as well as the clinical characteristics of age, T stage, N stage, and AJCC stage (Fig. 5A). The results demonstrated that the risk score could serve effectively as an independent prognostic factor. Following the multivariate analysis, age, AJCC stage, and risk score were found to be significant factors for prognosis. These variables were subsequently selected to construct a nomogram, facilitating the prediction of prognosis (Fig. 5B). The calibration curves were plotted to illustrate the satisfactory predictive consistency of the 1-, 3-, and 5-year survival rates by the nomogram. (Fig. 5C). The AUC values of 0.90, 0.80, and 0.75 at 1, 3, and 5 years, respectively, indicated that the nomogram could provide accurate predictions in the training set (Fig. 5D). In addition, to assess model applicability across breast cancer heterogeneity, we evaluated its performance in different PAM50 subtypes. The ROC curves showed that the AUC values for 1-year survival prediction were around 0.9 for all subtypes, and the AUC values for 5-year survival prediction remained above 0.7, supporting the robustness of the model across molecular subtypes (Supplementary Fig. 5). In the validation cohort, the performance of the nomogram was also assessed in predicting the 1-, 3-, and 5-year survival of breast cancer patients (Fig. 5E).

### Tumor-immune microenvironment analysis and effectiveness of a prognostic signature in predicting drug sensitivity

**Fig. 6:**
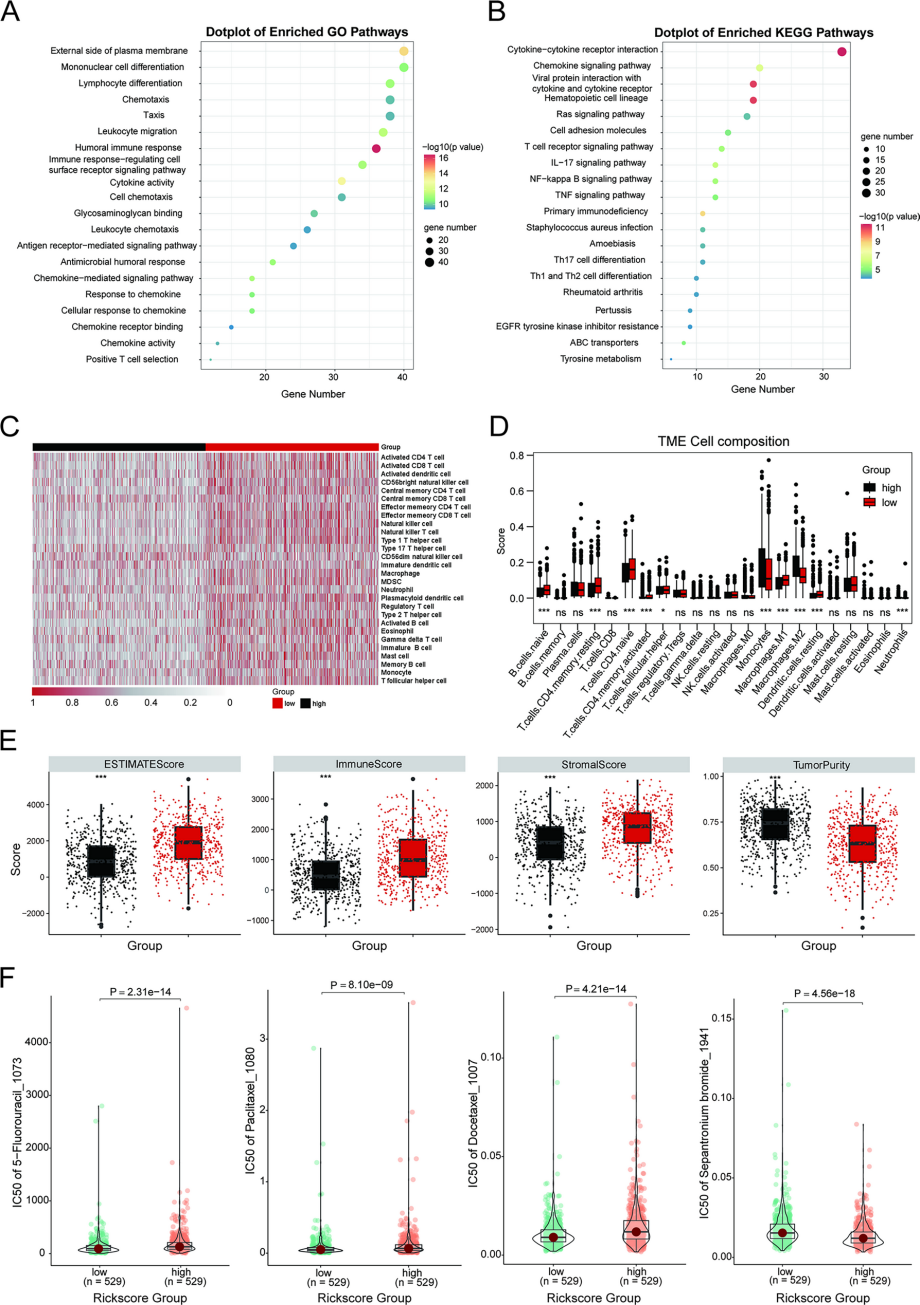
Tumor-immune microenvironment analysis and drug sensitivity prediction. **A**-**B** Gene Ontology (GO) and Kyoto Encyclopedia of Genes and Genomes (KEGG) enrichment analysis of DEGs between low- and high-risk score groups. **C** Heatmap of ssGSEA results for the two risk groups. **D** Boxplots illustrating differences in the infiltration levels of 22 immune cell types between the two risk groups. Statistics, Wilcoxon test. *p < 0.05; **p < 0.01; ***p < 0.001. **E** Differences in ESTIMATE score, stromal score, immune score, and tumor purity between the two risk groups. Statistics, Wilcoxon test. *p < 0.05; **p < 0.01; ***p < 0.001. **F** Boxplots comparing the IC50 values of drugs between high- and low-risk groups in the TCGA cohort. Statistics, Wilcoxon test. *p < 0.05; **p < 0.01; ***p < 0.001; ****p < 0.0001

The GO and KEGG enrichment analysis of DEGs between distinct risk groups revealed a predominant association with several immune responses and cytokine − cytokine receptor interaction (Fig. 6A-B). Meanwhile, the correlation with the external side of the plasma membrane and cell adhesion molecules highlighted the role of mechanical stimuli in the different risk groups. These results suggest differences in mechanical stimuli and immune responses between the two groups. In line with the survival benefit, patients in low-risk group demonstrated an increased prevalence of various infiltrating immune cells and a higher Immune Score with lower Tumor Purity, suggesting an immune “hot” phenotype (Fig. 6C-E). To investigate the correlation between the model and drug sensitivity, we calculated the predicted IC50 values for each drug in breast cancer patients. The landscape illustrates the correlation and significance between drug sensitivities and the risk score (Supplementary Fig. 6). We identified several drugs commonly used in clinical practice, such as 5-fluorouracil, palbociclib, and docetaxel, to exhibit a positive association between their IC50 values and risk scores (Fig. 6F). Conversely, the IC50 values of Sepantronium bromide, a prototypical survivin suppressant, exhibited a negative association with risk scores (Fig. 6F). These findings indicate that breast cancer patients classified as high-risk may demonstrate resistance to conventional chemotherapy treatments but may be sensitive to Sepantronium bromide. Therefore, Sepantronium bromide holds potential in therapeutic management of high-risk breast cancer patients.

### Mechanical stimulation impacts breast cancer prognosis by reshaping the immune microenvironment of the breast tumor

**Fig. 7:**
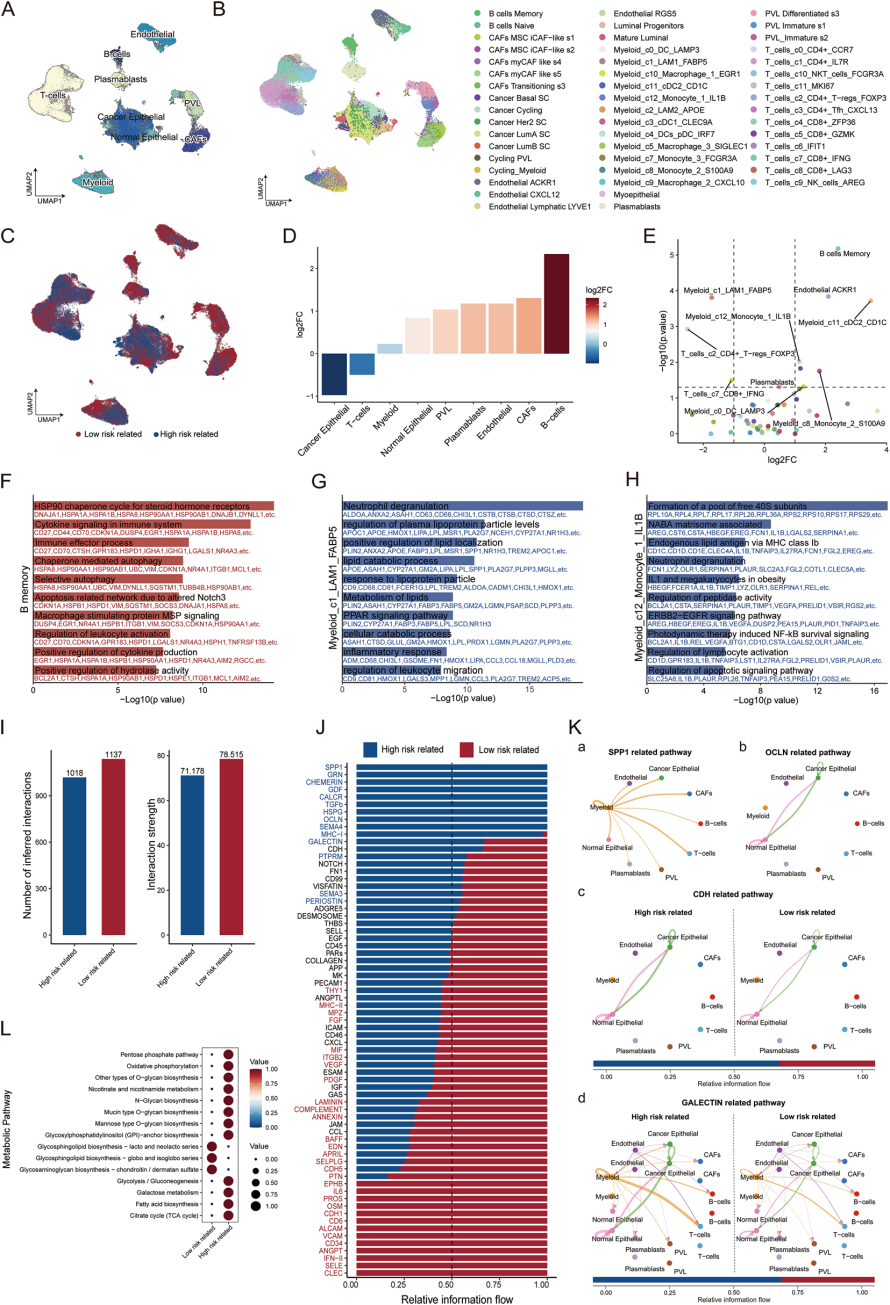
Mechanical stimulation influence breast cancer prognosis by altering the tumor’s immune microenvironment. **A**-**B** UMAP analysis identifying nine major cell types and forty-nine subtypes in breast cancer samples. **C** UMAP analysis of cells associated with SCISSOR-based risk groups. **D**-**E** Proportions of cells in different major cell types and subtypes associated with risk score. **F**–**H** Enrichment analysis of DEGs of memory B cells **F**, Monocyte_1_IL1B **G** and LAM1_FABP5 **H** between two risk-related groups. **I** Differences in the number of cells and the intensity of cellular communication associated with different risk scores. **J** Network analysis showing overall information flow differences between risk groups in the inferred cellular communication network. **K** Circos plots illustrating the SPP1, OCLN, CDH, and GALECTIN signaling pathways in related cells across different risk groups. **L** Dotplot showing metabolic scores across different risk groups

We next aimed to identify the cell types within the tumor microenvironment (TME) associated with distinct risk subgroup phenotypes. Processed single-cell sequencing data from 26 breast cancer patients were downloaded from the Broad Institute Single Cell portal (https://singlecell.broadinstitute.org/single_cell/study/SCP1039). The cells were clustered and annotated into nine major cell types and forty-nine subtypes (Fig. 7A-B).

To identify TME cell types associated with different risk phenotypes, we applied the SCISSOR algorithm in conjunction with RNA sequencing data from training set patients and their corresponding risk group classifications (Fig. 7C). In comparison to the high-risk group, the low-risk group exhibited a higher presence of B cells and fewer tumor cells (Fig. 7D). Specifically, the low-risk group showed an increased number of memory B cells, Endothelial_ACKR1, and Myeloid_cDC2_CD1C, alongside a decreased number of CD4 + T regulatory cells_FOXP3, Monocyte_1_IL1B, and LAM1_FABP5 (Fig. 7E). These cells exhibit differences across risk groups constructed based on characteristics related to the mechanical stimuli, suggesting a potential association with mechanical stimulation. Memory B cells can function as antigen-presenting cells, promoting the expansion of both memory and naive tumor-associated T cell responses, thereby enhancing anti-tumor immunity [42]. LAM1_FABP5 is believed to play an immune-suppressive role and is associated with poorer survival rates in breast cancer [43]. Further enrichment analysis was performed on cell subpopulations with significant differences in frequency using Metascape, the results revealed that memory B cells in both groups were significantly associated with immune effector processes (Fig. 7F) [44]. Monocyte_1_IL1B exhibited a strong correlation with mechanistically related pathways, such as the ERBB2 − EGFR signaling pathway, which has been reported to promote the proliferation and migration of breast cancer cells [45] (Fig. 7G). Further enrichment analysis of other cell types indicated significant associations with inflammatory responses (Fig. 7H, Supplementary Fig. 7). After the preliminary finding that mechanical stimuli may be associated with the frequency of immune cells, we aimed to further investigate whether it influences the crosstalk within the immune microenvironment. To this end, we employed CellChat to analyze intercellular communication through ligand-receptor signaling, with the goal of elucidating the cellular interactions that distinguish the high-risk and low-risk groups. The results showed that the number and strength of inferred interactions were greater in the low-risk group (Fig. 7I). Specifically, in the low-risk group, pathways related to inflammatory factors such as IFN-II and IL6 were enriched. While in the high-risk group, pathways related to mechanical stimuli, including secreted phosphoprotein 1 (SPP1), occludin (OCLN), cadherin (CDH), and galectin (GALECTIN) signaling, were enriched. These pathways play crucial roles in intercellular interactions, cell junctions, cell adhesion, and cell–matrix interactions (Fig. 7J-K). The low-risk group exhibits a stronger immune response, while the mechanical stimuli in the high-risk group may contribute to immune suppression. Metabolic scores for the different groups were calculated using scMetabolism. The results revealed that the metabolism of glycan biosynthesis, oxidative phosphorylation, and the pentose phosphate pathway were upregulated in the high-risk group, whereas glycosphingolipid and glycosaminoglycan biosynthesis were enhanced in the low-risk group (Fig. 7L).

### TEX19 affects the biological behaviors of breast cancer in vivo and in vitro

**Fig. 8:**
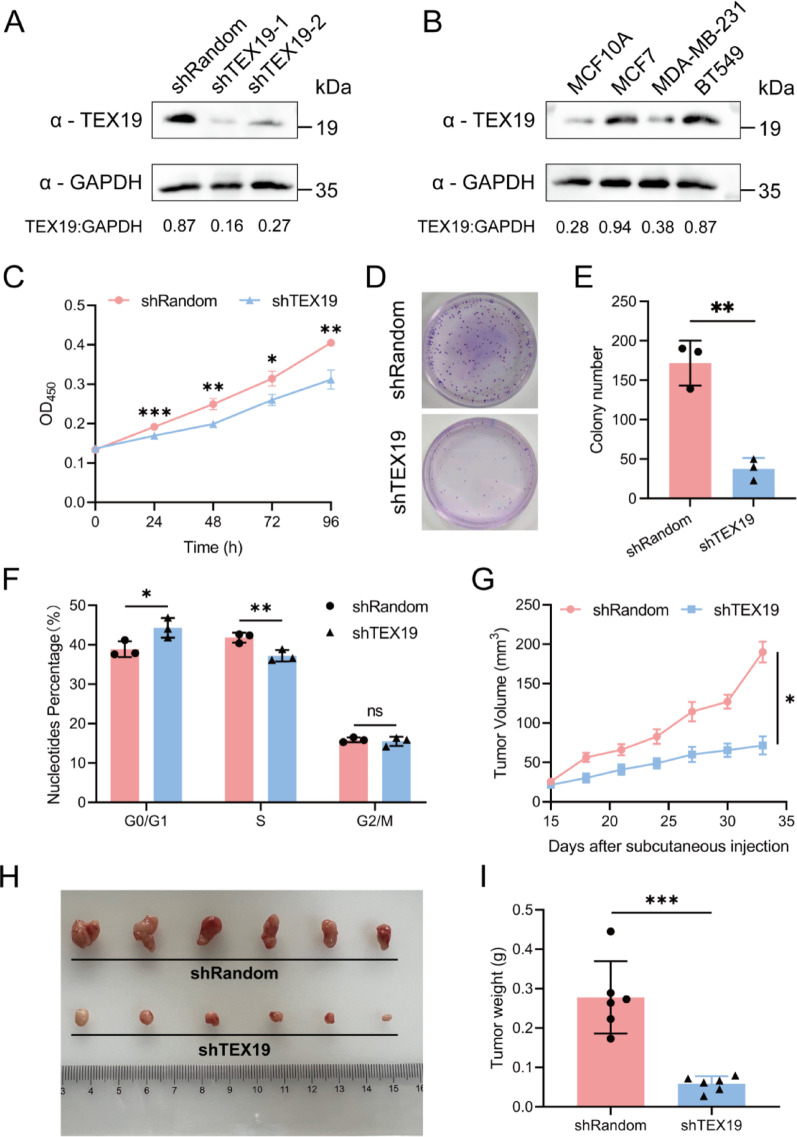
TEX19 affects the biological behaviors of breast cancer in vivo and invitro. **A** Expression levels of TEX19 and GAPDH were measured by Western blotting in TEX19 knockdown-treated (shTEX19) and control (shRandom) MCF-7 cells. **B** TEX19 protein levels in breast cancer cell lines (MCF-7, MDA-MB-231, BT-549) and the normal mammary epithelial cell line MCF-10A, measured by Western blotting. **C** Growth curve of TEX19 knockdown-treated and control (shRandom) MCF-7 cells. Results are presented as mean ± SD of triplicate experiments. Statistics, two-tailed Students’ t test. *p < 0.05; **p < 0.01; ***p < 0.001; ****p < 0.0001. **D**-**E** Colony formation assay in TEX19 knockdown-treated and control (shRandom) MCF-7 cells. The number of colonies formed was counted, and results are expressed as the mean ± SD of triplicate experiments. Statistics, two-tailed Students’ t test. *p < 0.05. **F** Flow cytometry analysis showing the effect of shTEX19 on the cell cycle in MCF-7 cells. The experiment was repeated three times. Statistics, two-way ANOVA. *p < 0.05; **p < 0.01. **G** Tumor sizes were measured from day 15. n = 6, Statistics, two-tailed Students’ t test. *p < 0.05. **H** Images illustrating breast tumor burdens in nude mice grafted with TEX19 knockdown-treated and control (shRandom) MCF-7 cells. n = 6. **I** Tumor weight at day 34 in nude mice. Data are presented as mean ± SD of six experiments and analyzed by unpaired Student’s t-test. *p < 0.05; **p < 0.01; ***p < 0.001; ****p < 0.0001

Testis Expressed 19 (TEX19) has been reported to be upregulated in various cancers, including bladder cancer and ovarian cancer, where it promotes tumor progression [46, 47]. Among the 15 model genes, TEX19 showed the highest hazard ratio (HR = 1.313) in univariate Cox regression, highlighting its potential significant impact on the prognosis of breast cancer (Supplementary Fig. 8). Furthermore, in our prognostic model, TEX19 was assigned one of the highest coefficient weights among all the genes. Single-cell transcriptome data further revealed that TEX19 is predominantly expressed in tumor cells (Supplementary Fig. 9), supporting its biological relevance in breast cancer progression. These computational and transcriptomic findings, together with prior evidence of TEX19’s oncogenic role, prompted us to prioritize it for functional validation. To further understand the role of TEX19 played in breast cancer, we generated a TEX19 Knockout MCF-7 cell line (Fig. 8A). Western blot analysis further confirmed that TEX19 protein was markedly upregulated in breast cancer cell lines (MCF-7, MDA-MB-231, BT-549) compared with the normal mammary epithelial cell line MCF-10A (Fig. 8B).We selected shTEX19-1, which had a better knockdown effect, for subsequent experiments. In proliferation analyses, shTEX19 cells lost capability to grow; however, shRandom cells sustained cells’ survival (Fig. 8C). Colonies analyses showed that TEX19 is essential for the growth of breast cancer (Fig. 8D-E). In addition, shTEX19 cells were arrested in G1 phase, which indicated that TEX19 is essential for tumor cell proliferation (Fig. 8F). In nude mice grafted with shTEX19 cancer cells, growth of tumors in mice was significantly impeded (Fig. 8G-I). These results taken together suggested that TEX19 is involved in the proliferation of breast cancer.

## Discussion

Our study is the first to construct a mechanical stimulus-related signature within the TCGA breast cohort and further validate its performance in an external cohort, METABRIC. We developed a nomogram that incorporates clinical characteristics along with a mechanical stimulus-related risk score with robust predictive performance. Additionally, we investigated the association between the mechanical stimulus-related risk score and the TIME, as well as drug sensitivity. Our findings suggest that mechanical stimuli may impact breast cancer prognosis by reshaping the immune microenvironment. Additionally, the mechanical stimulus-related signature could serve as a valuable tool to predict patient outcomes and guide personalized therapeutic strategies in breast cancer. In vivo and in vitro experiments using TEX19, the key model gene, knockout cells in nude mice demonstrated a significant reduction in tumor growth, confirming the involvement of TEX19 in breast cancer proliferation.

Mechanical stimuli have a significant impact on the biological behavior of cells and have attracted considerable attention from researchers in recent years. Tumor cells adapt to mechanical stresses within the primary tumor via mechanosensing and mechanotransduction mechanisms, particularly in the context of heightened matrix stiffness, thereby augmenting their fitness to proficiently navigate the subsequent obstacles encountered during the metastatic cascade [48]. We established a signature comprising 15 genes associated with mechanical stimuli (ALDH3A1, CCL19, CDH19, CEACAM5, CPLX2, CWH43, CXCL1, FABP7, JCHAIN, KIAA0319, KRT15, PIGR, SPIB, TCN1, TEX19) and demonstrated its predictive capability for overall survival in breast cancer patients. In advance, the nomogram, integrating age, AJCC stage, and risk score, showed promising predictive performance with area under the curve (AUC) values of 0.90, 0.80, and 0.75 at 1, 3, and 5 years, respectively. These findings suggest that the mechanical stimuli signature holds potential for delivering precise prognostic estimations for breast cancer patients.

TEX19 protein has been demonstrated to function as a cancer-testis antigen encoded by a gene specific to mammals, situated at 17q25.3 on human chromosome 17 [49]. Tex19.1 and Tex19.2 are duplication formations of Tex19 in rodents and preliminary analysis indicates that TEX19 expression orthologous to Tex19.1 is present in the adult testis and during the early stages of embryonic development. The expression of germline genes has been associated with poor prognosis in various cancers and TEX19 is essential for promoting cell proliferation in diverse cancer cell types, potentially through the regulation of oncogenic transcripts [50, 51]. For example, the expression of TEX19 is higher in high-grade bladder cancer tissues compared to low-grade bladder cancer, suggesting a potential role for TEX19 in the progression of bladder cancer [46]. TEX19 is also markedly upregulated in ovarian cancer, showing a correlation with advanced TNM stage, and knockdown of TEX19 results in the suppression of proliferation, migration, and invasion of ovarian cancer cells [47]. In this research, we found that TEX19, the key gene in the signature model, was associated with poor prognosis in breast cancer patients. The knockdown of TEX19 inhibited the proliferation of breast cancer cells both in vitro and in vivo. In line with our findings, another recent study found that TEX19 promoted the progression of breast cancer by modulating SKP2-mediated ubiquitination of CDK4 [52]. These findings underscore the significant role that TEX19 plays in promoting the progression of breast cancer.

Many biophysical properties of tissues shape immune cell function through mechanical stimulation signal transduction, activating immune cells and driving their effector functions [14]. Growing evidence suggests that both cellular and acellular components within the TME, particularly immune cells, have the capacity to reprogram tumor initiation, progression, invasion, metastasis, and treatment outcomes [53]. The low-risk group was categorized as exhibiting a "hot" immunophenotype linked to survival benefits, whereas the high-risk group displayed the opposite effect, which was similar to different cluster groups. When it comes to specific immune cell types, CD8^+^ T cells were a notable component of the low-risk group. Previous studies have highlighted the role of CD8^+^ T cells in impeding tumor growth, enhancing immune responses, and supporting the efficacy of immunotherapy [54]. However, type 2 macrophages (M2) had the potential to create immunological barriers that impede CD8 ^+^ T cell-mediated antitumor immune responses [54]. The high-risk group exhibited a notable abundance of infiltrating macrophage M0 and M2 cell populations. M0 cells were regarded as quiescent macrophages capable of differentiating into two distinct phenotypes: M1 cells, linked to pro-inflammatory responses, and M2 cells, associated with anti-inflammatory responses [55]. The increased presence of M2 cells has been associated with tumor advancement and adverse cancer prognosis [34, 56], aligning with the findings of our study. Furthermore, the GO and KEGG enrichment analyses highlighted a significant correlation with humoral immune response, Cytokine − cytokine receptor interaction, and external side of plasma membrane, underscoring the importance of mechanical stimuli within the tumor microenvironment. The "hot" immune phenotype was demonstrated to significantly benefit from immunotherapy [57], a finding corroborated by drug sensitivity predictions. Our investigation revealed that drugs frequently employed in clinical settings, including 5-fluorouracil, palbociclib, and docetaxel, displayed elevated IC50 values in the high-risk group. However, The IC50 values of Sepantronium bromide showed an inverse correlation with risk scores, which suggested that breast cancer patients identified as high-risk may display resistance to traditional chemotherapy regimens but could potentially be responsive to Sepantronium bromide. Sepantronium bromide (YM155) is a prototypical survivin suppressant, which lead to the separation of the paraspeckle regulatory 54-kDa nuclear RNA-binding protein (p54nrb) from interleukin enhancer-binding factor 3 (ILF3), resulting in downregulation of surviving [58]. For breast cancer, Sepantronium bromide can cause reduction in cellular proliferation, induce spontaneous apoptosis and decrease spontaneous metastases in human triple-negative breast cancer [59]. However, in a phase II trial, Sepantronium bromide exhibited poor performance in HER2-negative metastatic breast cancer [60], indicating the necessity for further research to elucidate the potential role of this drug in cancer therapy.

From the analysis results of single cells, it can be observed that the high-risk group had a lower proportion of memory B cells and Endothelial_ACKR1 cells and a higher proportion of CD4^+^ T regulatory cells_FOXP3 and LAM1_FABP5 cells. Memory B cells were known to promote anti-tumor immunity by acting as antigen-presenting cells, while Endothelial_ACKR1 cells were associated with better prognosis in some tumors; CD4^+^ T regulatory cells_FOXP3 mediated peripheral immune tolerance and suppressed cellular immunity, including anti-tumor responses, whereas LAM1_FABP5 cells, which played an immune-suppressive role, were linked to poorer survival rates in breast cancer [42, 43, 61, 62]. Our results suggested that these cell types may be associated with mechanical stimulation-related features and could be of interest in immunotherapy for high-risk patients. Additionally, mechanical stimulus-related signaling pathways such as SPP1 and galectin were enhanced, as well as metabolic pathways related to mechanical stimuli, such as glycan biosynthesis. These results suggested that factors related to mechanical stimuli may influence the proportion of key cell subpopulations in the breast cancer microenvironment, modulate the strength of intercellular communication, and affect certain metabolic pathways, thereby leading to prognostic-related phenotypic changes. In line with the bulk analysis results, the overall findings indicated that mechanical stimulation may affect breast cancer prognosis by remodeling the immune microenvironment.

However, our study had several limitations. First, it relied on a public database, necessitating further validation through multicenter randomized controlled trials. Second, the inclusion of 15 key genes in the signature may have increased the workload, and there could be unidentified interactions between genes and their products, potentially affecting the signature’s efficacy. Third, while we successfully validated the prognostic signature in two independent GEO cohorts (GSE96058 and GSE20685), the lack of comprehensive clinical annotations in these datasets prevented us from assessing the full prognostic model. Therefore, future studies with more complete clinical information will be required to fully confirm its applicability. Fourth, given the well-recognized heterogeneity of breast cancer, our preliminary subgroup analyses provide only an initial exploration, and more detailed investigations are warranted to clarify subtype-specific prognostic implications. Finally, the drug sensitivity results were based on database data and have not been validated through cell experiments. Grouping cells according to risk scores proved challenging, and in vivo experiments could be conducted if necessary.

## Conclusion

In summary, this study, which utilized single-cell RNA sequencing and bulk RNA sequencing analysis, presents a pioneering prognostic signature incorporating MSRGs in breast cancer, with particular emphasis on mechanical stimuli that may influence breast cancer prognosis by remodeling the immune microenvironment. In vivo and in vitro experiments further validate the critical role of the key model gene TEX19 in driving breast cancer progression. These findings offer a novel approach to clinical management, providing valuable insights into the role of mechanical cues in breast tumor biology and highlighting their potential for enhancing prognostic accuracy and informing personalized treatment strategies.

## Supplementary Information

Supplementary file1.

Supplementary file2.

Supplementary file3.

## Data Availability

The datasets are available from The Cancer Genome Atlas (TCGA), the Broad Institute Single Cell portal (https://singlecell.broadinstitute.org/single_cell/study/SCP1039), and the Molecular Signature Database (MSigDB) (http://www.gseamsigdb.org/gsea/index.jsp), or data availability part of the corresponding articles. All data and code necessary for the analyses are available upon request. For further information, please contact the corresponding author.
