# Supplementary material for: Integrating bulk and single-cell RNA sequencing analysis to reveal characterization of mechanical stimulus-related genes and prognostic signatures in breast cancer
Source: Breast Cancer Res. 2025 Nov 13;27:204. doi: 10.1186/s13058-025-02130-6 (PMC12616973; doi:10.1186/s13058-025-02130-6)
Supplement: Supplementary file 1 — Supplementary file1. [file 13058_2025_2130_MOESM1_ESM.docx]

**
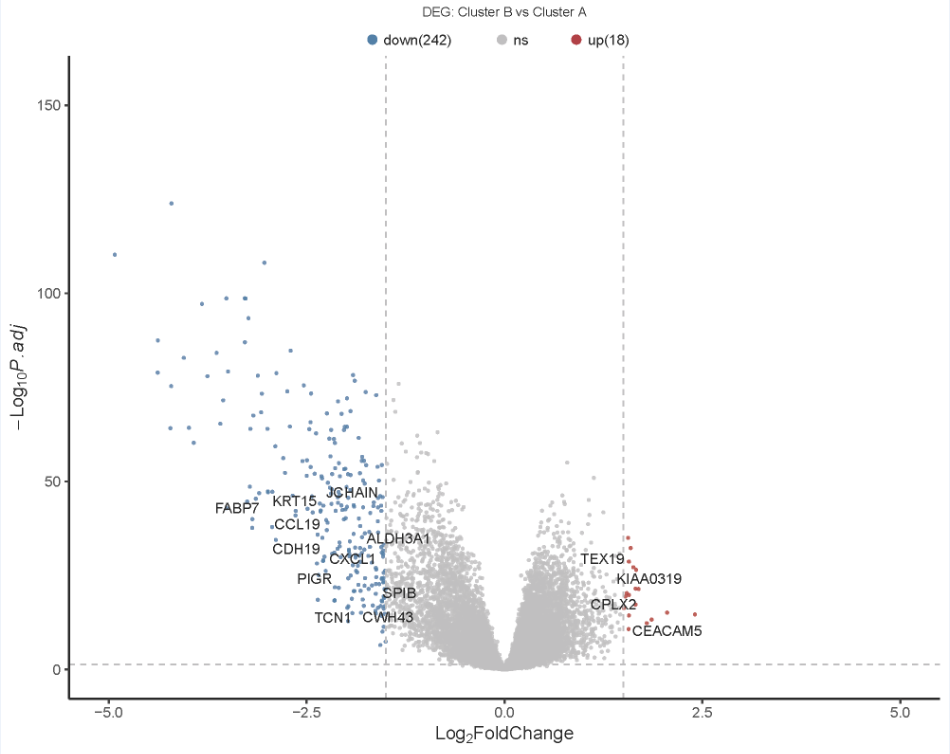
**

**Supplementary Fig. 1 |** **Differential expressed genes (DEGs) between Cluster B and Cluster A.** A volcano map to exhibit DEGs between Cluster B and Cluster A, 15 model genes were highlighted on the plot.


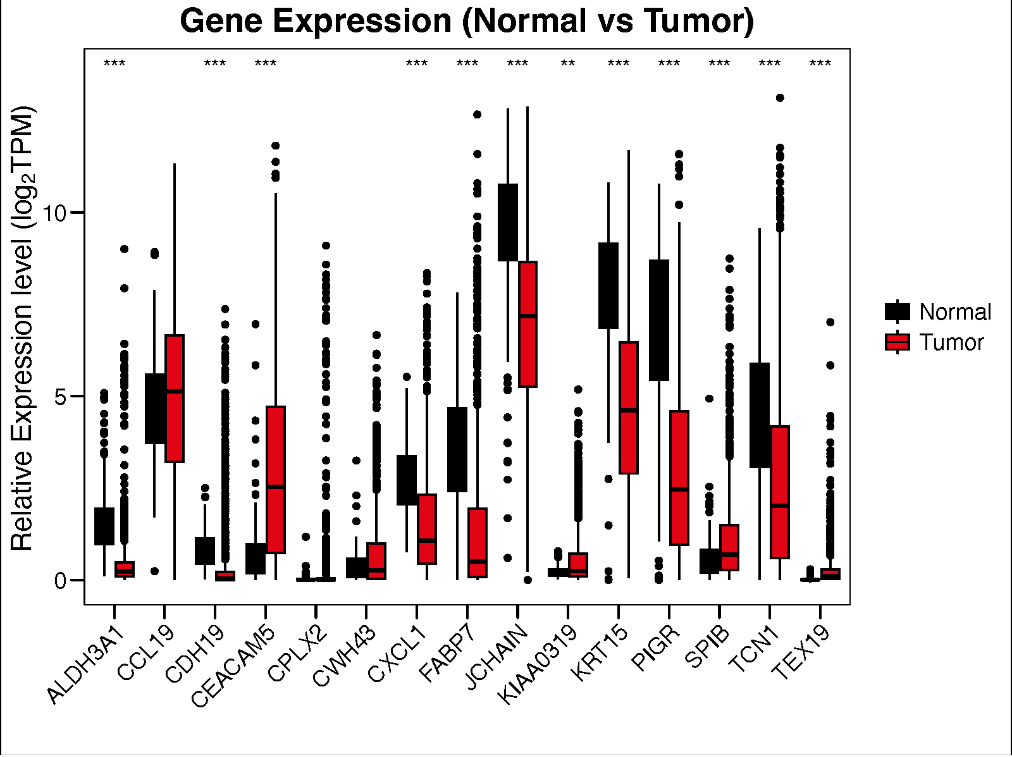


**Supplementary Fig. 2 | Differential expression of the 15 prognostic genes in TCGA.**
Box plots showing the expression of the 15 mechanical stimulus-related genes between breast cancer tissues and adjacent normal tissues in the TCGA cohort.


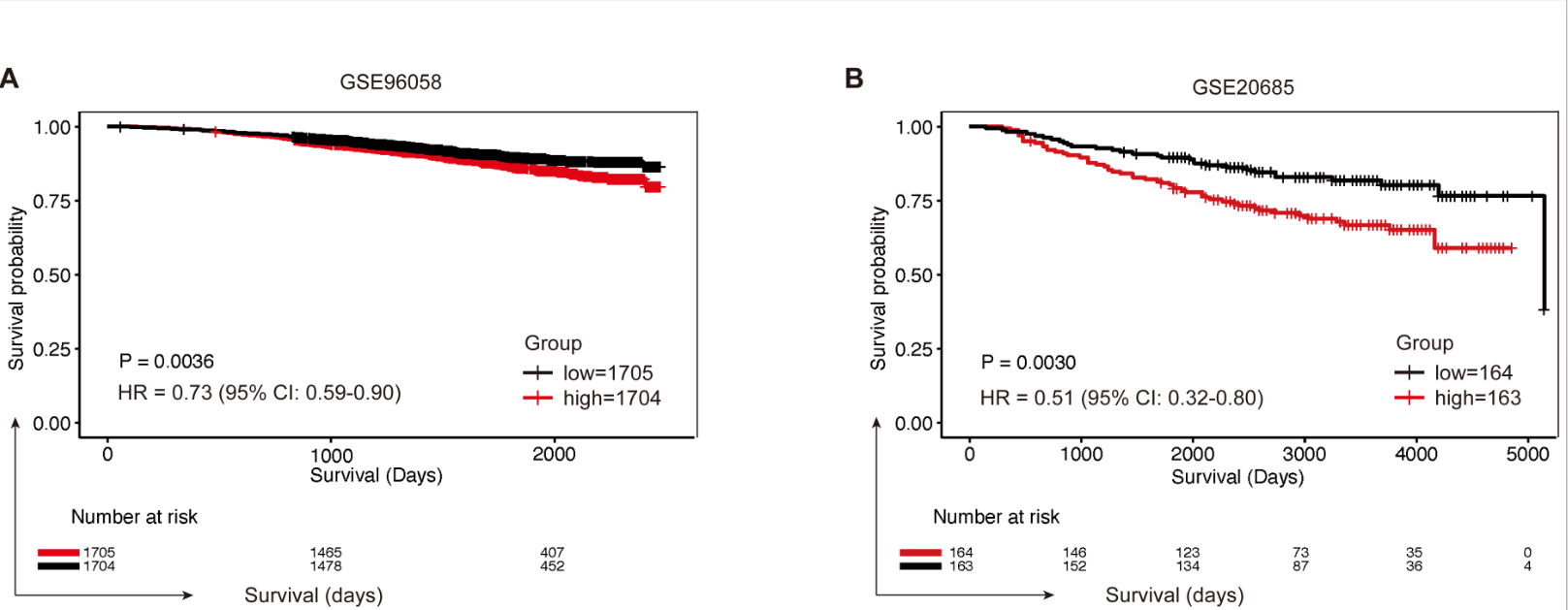


**Supplementary Fig. 3 | Kaplan–Meier survival analyses of the prognostic signature across independent cohorts.**
Kaplan–Meier curves showing overall survival in the GSE96058 and GSE20685 cohorts, with low-risk patients exhibiting significantly longer survival.


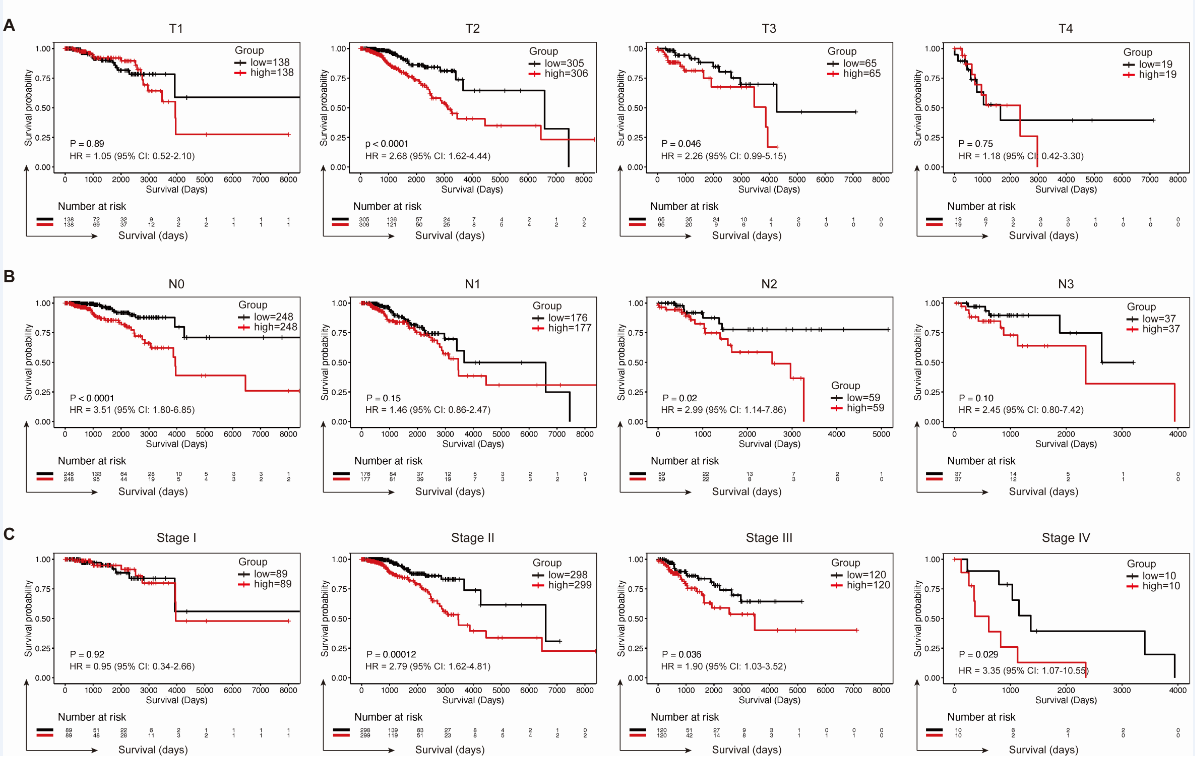


**Supplementary Fig. 4 | Subgroup survival analyses of the prognostic signature in the TCGA cohort.**
Kaplan–Meier curves of overall survival stratified by T stage (T1–T4), N stage (N0–N3), and clinical stage (I–IV), showing significantly better outcomes in the low-risk group for several early to intermediate subgroups and a consistent trend toward improved survival in other strata.


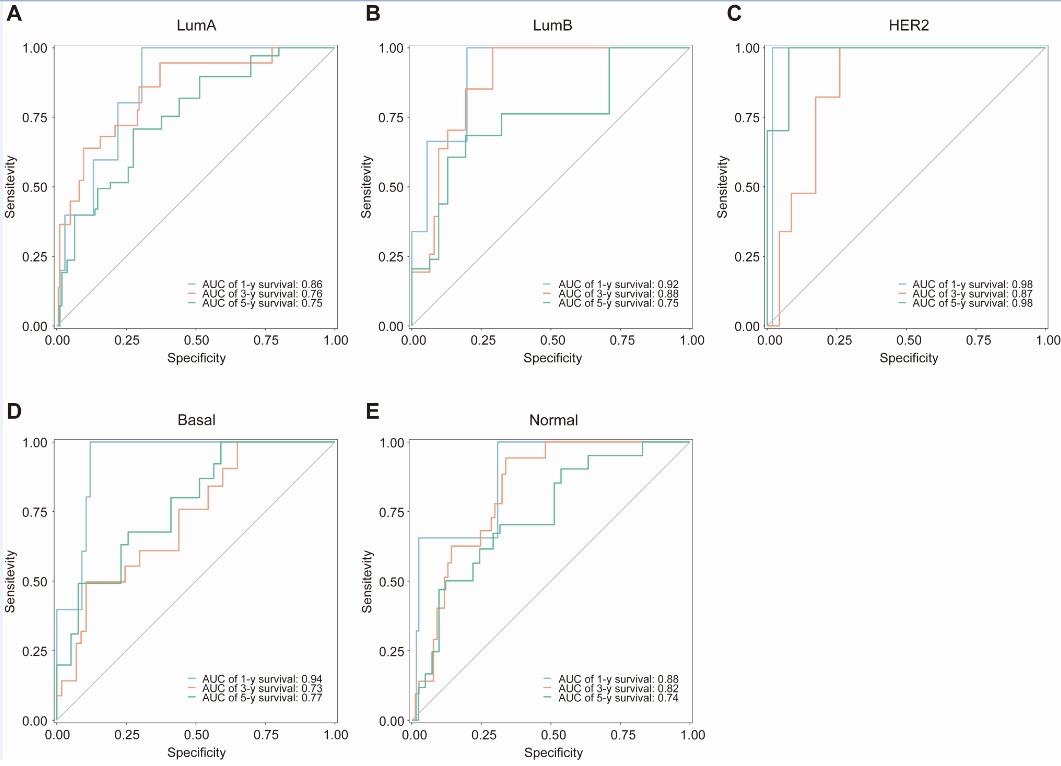


**Supplementary Fig. 5 | Prognostic performance of the signature across PAM50 subtypes.**
ROC curves showing 1-, 3-, and 5-year survival prediction based on the prognostic signature in different PAM50 breast cancer subtypes, demonstrating stable performance across molecular subtypes.


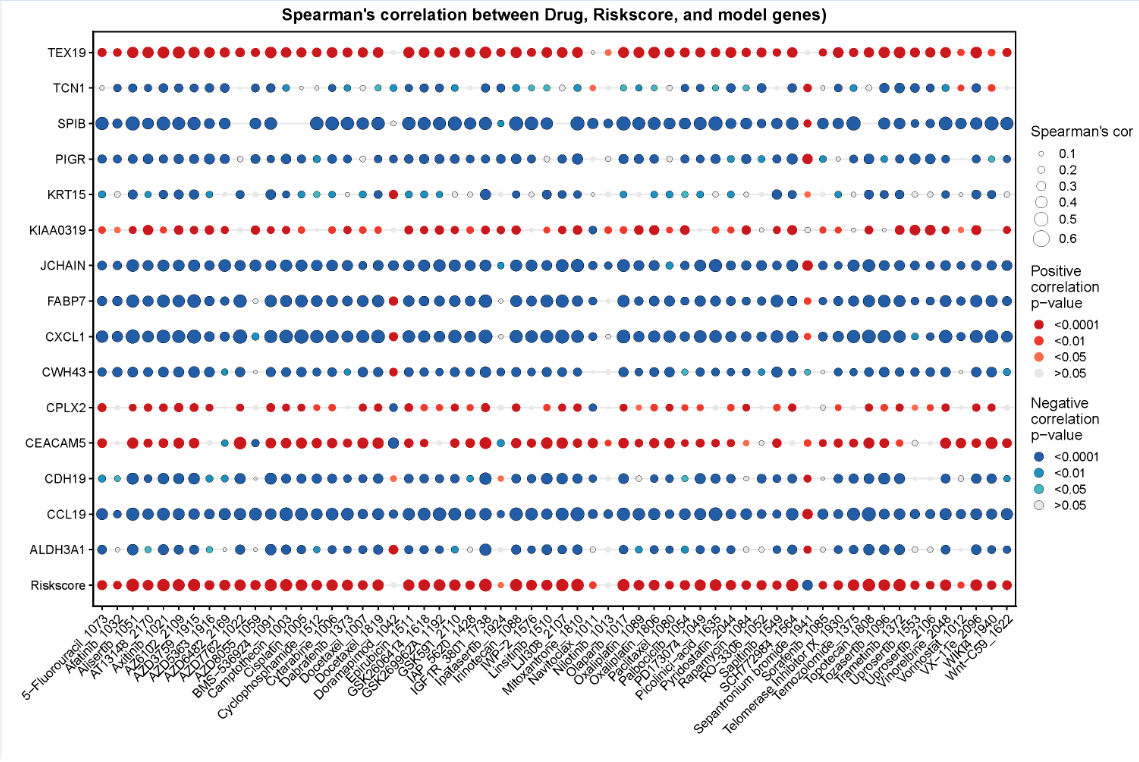


**Supplementary Fig. 6 | Efficacy of mechanical stimulus signature in predicting drug sensitivity.** Bubble plot of the relationship between drugs, risk scores, and model genes.


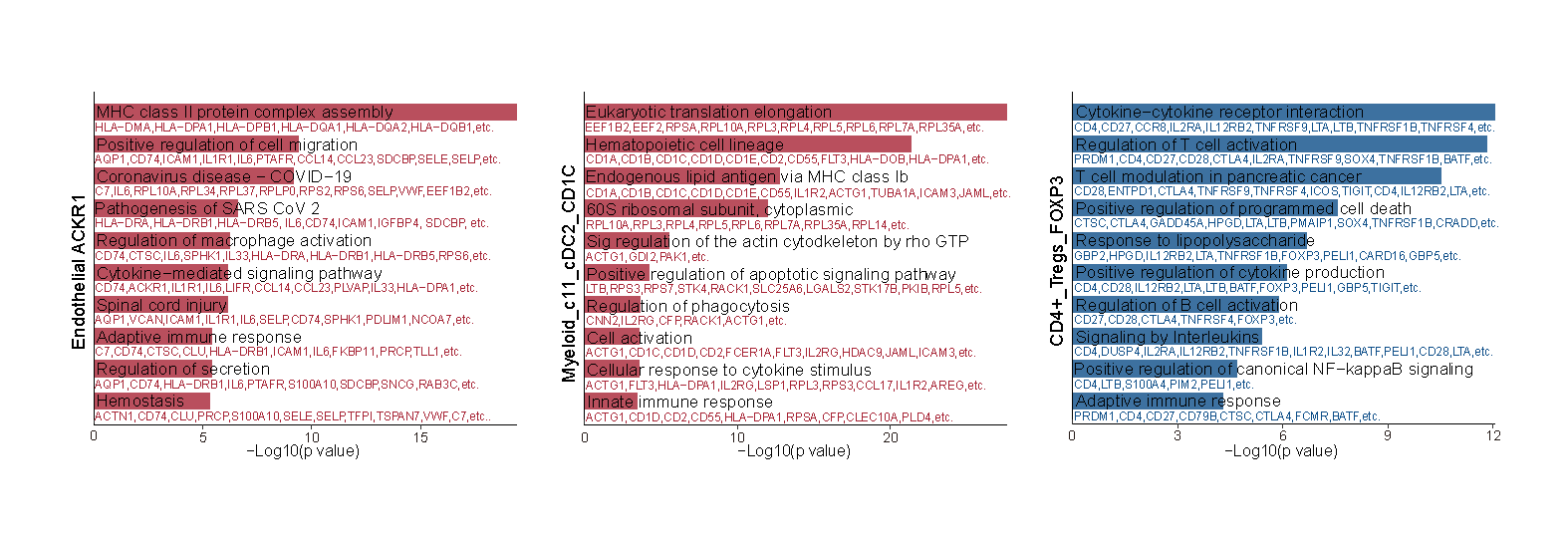


**Supplementary Fig. 7**| **Enrichment analysis of DEGs of** **Endothelial_ACKR1, Myeloid_cDC2_CD1C and CD4^+^ T regulatory cells_FOXP3 between two risk-related groups.**


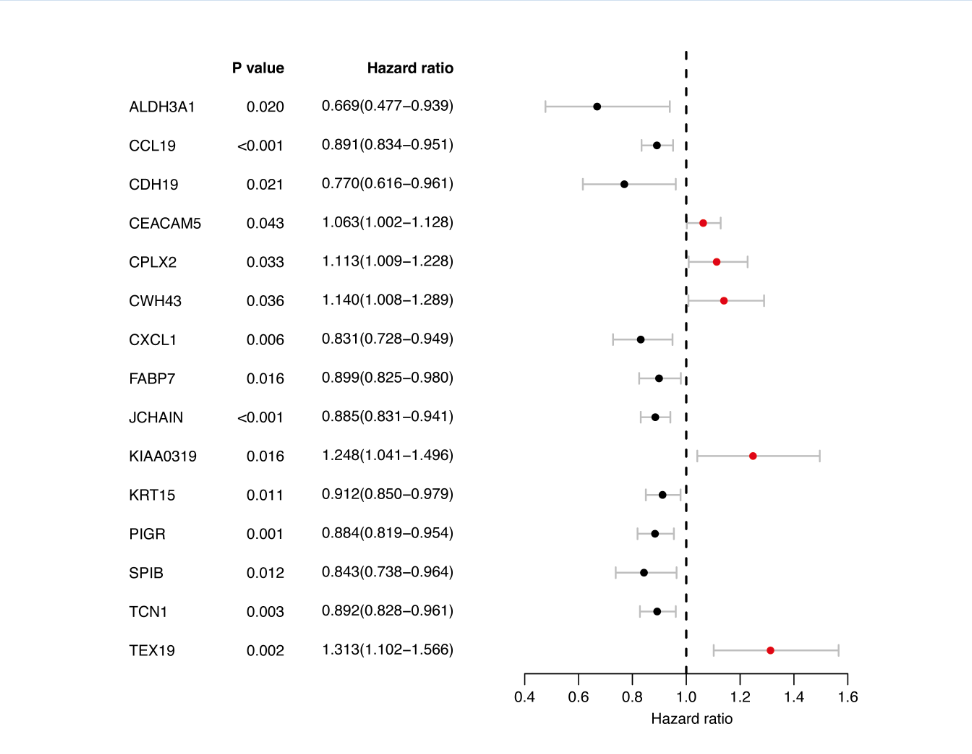


**Supplementary Fig. 8 | Forest plot of 15 mechanical stimulus-related genes included in the prognostic model.**

A forest plot displaying hazard ratios (HRs) and 95% confidence intervals for the 15 mechanical stimulus-related genes incorporated into the prognostic model.


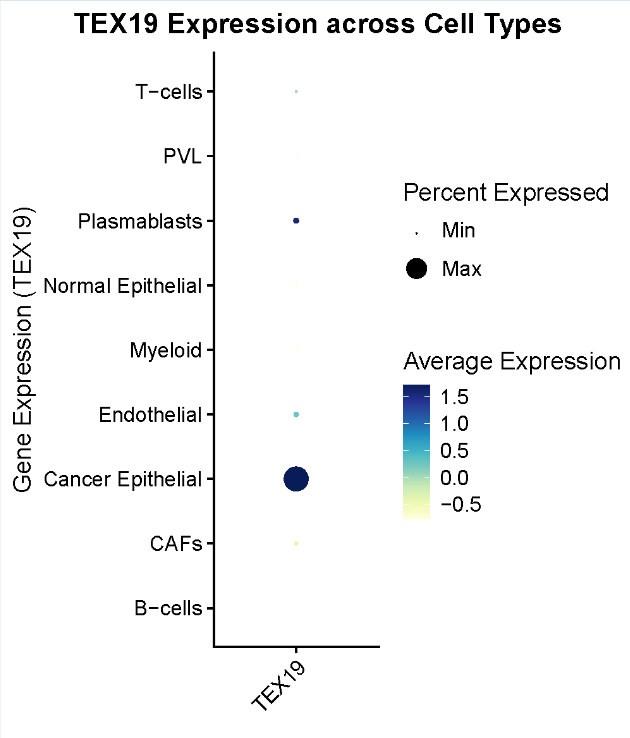


**Supplementary Fig. 9**| **Expression of TEX19 in Subpopulations of Breast Cancer.**

Dot plot showing the expression of TEX19 across major cell types in breast cancer.
