# Supplementary material for: Integrating bulk and single-cell RNA sequencing analysis to reveal characterization of mechanical stimulus-related genes and prognostic signatures in breast cancer
Source: Breast Cancer Res. 2025 Nov 13;27:204. doi: 10.1186/s13058-025-02130-6 (PMC12616973; doi:10.1186/s13058-025-02130-6)
Supplement: Supplementary file 2 — Supplementary file2. [file 13058_2025_2130_MOESM2_ESM.docx]

**Supplementary table 1 | Summary of clinical information from the TCGA and METABRIC**

| **Characteristic** | **TCGA** | **METABRIC** |
| --- | --- | --- |
| **Sample** | 1058 | 1980 |
| **Age (mean (SD)),**  **years** | 58.14 (12.91) | 61.09 (12.95) |
| **Time (mean (SD)),**  **days** | 1250.34 (1190.70) | 3756.70 (2283.76) |
| **Event (%)** |  |  |
| Alive | 911 (86.1) | 837 ( 42.3) |
| Dead | 147 (13.9) | 1143 ( 57.7) |
| **Stage (%)** |  |  |
| I | 178 (16.8) | 501 ( 25.3) |
| II | 597 (56.4) | 825 ( 41.7) |
| III | 240 (22.7) | 118 ( 6.0) |
| IV | 20 ( 1.9) | 10 ( 0.5) |
| NA | 23 ( 2.2) | 526 ( 26.6) |
| **T (%)** |  |  |
| 1 | 276 (26.1) | / |
| 2 | 611 (57.8) | / |
| 3 | 130 (12.3) | / |
| 4 | 38 ( 3.6) | / |
| NA | 3 ( 0.3) | / |
| **N (%)** |  |  |
| 0 | 496 (46.9) | / |
| 1 | 353 (33.4) | / |
| 2 | 118 (11.2) | / |
| 3 | 74 ( 7.0) | / |
| NA | 17 ( 1.6) | / |
| **Pam50 (%)** |  |  |
| Basal | 169 (16.0) | / |
| Her2 | 68 ( 6.4) | / |
| LumA | 494 (46.7) | / |
| LumB | 181 (17.1) | / |
| Normal | 134 (12.7) | / |
| NA | 12 ( 1.1) | / |

**Supplementary table 2 | 212 genes linked to the response to mechanical stimulus enrolled in this study**

| No. | Gene |
| --- | --- |
| 1 | ABHD12 |
| 2 | ACTA1 |
| 3 | ADGRV1 |
| 4 | ANGPT2 |
| 5 | ANKRD1 |
| 6 | ANKRD23 |
| 7 | ANO3 |
| 8 | AQP1 |
| 9 | ASIC2 |
| 10 | ASIC3 |
| 11 | ATAT1 |
| 12 | ATOH7 |
| 13 | ATP1A2 |
| 14 | ATP8A2 |
| 15 | BACE1 |
| 16 | BAD |
| 17 | BAG3 |
| 18 | BAK1 |
| 19 | BCL10 |
| 20 | BDKRB1 |
| 21 | BGLAP |
| 22 | BMP6 |
| 23 | BNIP3 |
| 24 | BTG2 |
| 25 | CAPN2 |
| 26 | CASP1 |
| 27 | CASP2 |
| 28 | CASP5 |
| 29 | CASP8 |
| 30 | CASP8AP2 |
| 31 | CAV3 |
| 32 | CD40 |
| 33 | CDH2 |
| 34 | CHEK1 |
| 35 | CHI3L1 |
| 36 | CHRNA10 |
| 37 | CHRNA9 |
| 38 | CITED2 |
| 39 | CLCN6 |
| 40 | CNN2 |
| 41 | CNTNAP2 |
| 42 | COL11A1 |
| 43 | COL1A1 |
| 44 | CRADD |
| 45 | CSRP3 |
| 46 | CTNNB1 |
| 47 | CXCL10 |
| 48 | CXCL12 |
| 49 | CXCR4 |
| 50 | DAG1 |
| 51 | DCANP1 |
| 52 | DDR2 |
| 53 | DMD |
| 54 | DRD2 |
| 55 | EDN1 |
| 56 | ENG |
| 57 | ETV1 |
| 58 | F11R |
| 59 | FADD |
| 60 | FAS |
| 61 | FGF2 |
| 62 | FOS |
| 63 | FOSB |
| 64 | FOSL1 |
| 65 | FYN |
| 66 | GADD45A |
| 67 | GATA4 |
| 68 | GCLC |
| 69 | GDF5 |
| 70 | GPI |
| 71 | GSN |
| 72 | HABP4 |
| 73 | HPN |
| 74 | HTR2A |
| 75 | HTT |
| 76 | IGF1R |
| 77 | IGFBP2 |
| 78 | IHH |
| 79 | IL13 |
| 80 | IL1B |
| 81 | IL33 |
| 82 | IRF1 |
| 83 | ITGA2 |
| 84 | ITGB3 |
| 85 | JUP |
| 86 | KCNA1 |
| 87 | KCNA5 |
| 88 | KCNC1 |
| 89 | KCNJ2 |
| 90 | KCNK2 |
| 91 | KCNK4 |
| 92 | KCNQ1 |
| 93 | KCNQ3 |
| 94 | KIAA0319 |
| 95 | KIT |
| 96 | KRT5 |
| 97 | LARGE1 |
| 98 | LHFPL5 |
| 99 | LRP11 |
| 100 | LTBR |
| 101 | MAG |
| 102 | MAP1B |
| 103 | MAP2K4 |
| 104 | MAP3K1 |
| 105 | MAP3K14 |
| 106 | MAP3K2 |
| 107 | MAPK14 |
| 108 | MAPK3 |
| 109 | MAPK8 |
| 110 | MBD2 |
| 111 | MDK |
| 112 | MEIS2 |
| 113 | MKKS |
| 114 | MMP14 |
| 115 | MMP2 |
| 116 | MPO |
| 117 | MTPN |
| 118 | MYD88 |
| 119 | NEUROG1 |
| 120 | NFKB1 |
| 121 | NFKBIA |
| 122 | NPPA |
| 123 | NRXN1 |
| 124 | NRXN2 |
| 125 | NTRK1 |
| 126 | P2RX3 |
| 127 | P2RX7 |
| 128 | P2RY1 |
| 129 | PDE2A |
| 130 | PDZD7 |
| 131 | PHF24 |
| 132 | PIEZO1 |
| 133 | PIEZO2 |
| 134 | PIK3CA |
| 135 | PJVK |
| 136 | PKD1 |
| 137 | PKD1L1 |
| 138 | PKD1L3 |
| 139 | PKD2 |
| 140 | PKD2L1 |
| 141 | PKD2L2 |
| 142 | PKDREJ |
| 143 | PLEC |
| 144 | POSTN |
| 145 | PPL |
| 146 | PSPH |
| 147 | PTCH1 |
| 148 | PTGER4 |
| 149 | PTGS2 |
| 150 | PTK2 |
| 151 | PTK2B |
| 152 | PTN |
| 153 | RAF1 |
| 154 | RELA |
| 155 | RETN |
| 156 | RPS6KB1 |
| 157 | RYR2 |
| 158 | SCEL |
| 159 | SCN11A |
| 160 | SCN1A |
| 161 | SCN9A |
| 162 | SCX |
| 163 | SERPINE2 |
| 164 | SHANK3 |
| 165 | SLC1A3 |
| 166 | SLC26A5 |
| 167 | SLC2A1 |
| 168 | SLC38A2 |
| 169 | SLC8A1 |
| 170 | SLC9A1 |
| 171 | SLITRK6 |
| 172 | SOST |
| 173 | SOX9 |
| 174 | SRC |
| 175 | STAT1 |
| 176 | STRA6 |
| 177 | STRBP |
| 178 | STRC |
| 179 | SUN1 |
| 180 | TACR1 |
| 181 | TCAP |
| 182 | THBS1 |
| 183 | TIFAB |
| 184 | TLR3 |
| 185 | TLR4 |
| 186 | TLR5 |
| 187 | TLR7 |
| 188 | TLR8 |
| 189 | TMC1 |
| 190 | TMC2 |
| 191 | TMEM120A |
| 192 | TMEM150C |
| 193 | TMEM87A |
| 194 | TNC |
| 195 | TNF |
| 196 | TNFRSF10A |
| 197 | TNFRSF10B |
| 198 | TNFRSF11A |
| 199 | TNFRSF1A |
| 200 | TNFRSF8 |
| 201 | TNFSF14 |
| 202 | TRPA1 |
| 203 | TRPV4 |
| 204 | TTN |
| 205 | TUBA1A |
| 206 | TXNIP |
| 207 | UCN |
| 208 | USP53 |
| 209 | WHRN |
| 210 | WNT11 |
| 211 | XPA |
| 212 | XPC |

**Supplementary table 3 | 260 DEGs between cluster A and cluster B**

| Gene | logFC | P.Value | adj.P.Val | DEG |
| --- | --- | --- | --- | --- |
| KRT5 | -5.11687 | 2.75E-160 | 5.37E-156 | Down |
| KRT17 | -4.20747 | 1.30E-128 | 1.27E-124 | Down |
| KRT14 | -4.92283 | 8.34E-115 | 5.43E-111 | Down |
| FAT2 | -3.03318 | 1.50E-112 | 7.30E-109 | Down |
| SFRP1 | -3.51286 | 6.72E-103 | 2.18E-99 | Down |
| TRIM29 | -3.27104 | 8.23E-103 | 2.29E-99 | Down |
| ANXA8L1 | -3.2802 | 5.87E-103 | 2.18E-99 | Down |
| DSC3 | -3.82161 | 2.67E-101 | 6.50E-98 | Down |
| ANXA8 | -3.23449 | 1.90E-97 | 4.11E-94 | Down |
| DSG3 | -4.37933 | 1.71E-91 | 3.34E-88 | Down |
| KLK8 | -3.27973 | 5.84E-91 | 1.04E-87 | Down |
| MRGPRX3 | -2.70093 | 1.10E-88 | 1.79E-85 | Down |
| EDN3 | -3.63672 | 4.47E-88 | 6.71E-85 | Down |
| KLK7 | -4.05084 | 9.82E-87 | 1.37E-83 | Down |
| DSG1 | -3.49191 | 4.76E-83 | 6.19E-80 | Down |
| KRT6B | -4.3814 | 1.00E-82 | 1.23E-79 | Down |
| CLDN19 | -2.88111 | 1.45E-82 | 1.67E-79 | Down |
| LAMB3 | -1.9131 | 4.86E-82 | 5.27E-79 | Down |
| BBOX1 | -3.11595 | 7.59E-82 | 7.79E-79 | Down |
| KLK6 | -3.75224 | 1.09E-81 | 1.07E-78 | Down |
| NSG1 | -1.89201 | 1.91E-80 | 1.77E-77 | Down |
| CLCA4 | -2.53497 | 3.49E-79 | 2.96E-76 | Down |
| KLK5 | -4.21025 | 5.62E-79 | 4.57E-76 | Down |
| ECRG4 | -2.74337 | 1.45E-77 | 1.13E-74 | Down |
| IL34 | -1.75347 | 2.35E-77 | 1.76E-74 | Down |
| OSR1 | -2.44342 | 5.79E-77 | 4.19E-74 | Down |
| SMYD1 | -3.06547 | 6.90E-77 | 4.81E-74 | Down |
| CX3CL1 | -1.61989 | 1.74E-76 | 1.17E-73 | Down |
| TSLP | -1.98986 | 1.27E-75 | 8.29E-73 | Down |
| KLK10 | -3.55392 | 4.69E-75 | 2.86E-72 | Down |
| NGFR | -2.10346 | 8.96E-75 | 5.30E-72 | Down |
| TEPP | -1.9451 | 3.56E-72 | 2.04E-69 | Down |
| COL17A1 | -3.0746 | 7.82E-72 | 4.24E-69 | Down |
| PCARE | -2.24286 | 1.51E-71 | 7.98E-69 | Down |
| TACR1 | -2.05787 | 2.05E-71 | 1.05E-68 | Down |
| SERPINB5 | -3.17278 | 6.08E-71 | 3.04E-68 | Down |
| TP63 | -2.44999 | 3.88E-69 | 1.89E-66 | Down |
| ROPN1 | -3.58993 | 9.51E-69 | 4.52E-66 | Down |
| SAA1 | -2.7102 | 5.45E-68 | 2.53E-65 | Down |
| FMO2 | -2.01749 | 6.72E-68 | 3.05E-65 | Down |
| CNTNAP3 | -1.98936 | 6.95E-68 | 3.08E-65 | Down |
| STAC2 | -3.98634 | 1.29E-67 | 5.60E-65 | Down |
| SOX10 | -4.22041 | 1.70E-67 | 7.20E-65 | Down |
| SYT8 | -2.9938 | 2.51E-67 | 1.04E-64 | Down |
| FGFBP1 | -3.20949 | 2.63E-67 | 1.07E-64 | Down |
| PAK5 | -2.46601 | 3.21E-67 | 1.28E-64 | Down |
| RNASE7 | -2.19004 | 5.44E-67 | 2.12E-64 | Down |
| CRYAB | -2.02752 | 5.64E-67 | 2.16E-64 | Down |
| IRX1 | -2.38017 | 4.53E-66 | 1.67E-63 | Down |
| ID4 | -1.84218 | 7.69E-65 | 2.73E-62 | Down |
| EDAR | -2.21262 | 1.25E-64 | 4.37E-62 | Down |
| OVCH2 | -2.15523 | 1.47E-64 | 5.04E-62 | Down |
| GABRP | -3.92555 | 1.61E-63 | 5.41E-61 | Down |
| CDH22 | -2.1426 | 1.82E-63 | 6.03E-61 | Down |
| GPR12 | -2.89546 | 1.47E-62 | 4.61E-60 | Down |
| CHI3L1 | -2.198 | 7.18E-60 | 2.09E-57 | Down |
| SYNM | -1.7977 | 1.02E-59 | 2.92E-57 | Down |
| SOSTDC1 | -2.79414 | 2.31E-59 | 6.45E-57 | Down |
| SPHKAP | -2.49537 | 8.72E-59 | 2.40E-56 | Down |
| RNF186 | -1.8033 | 1.19E-58 | 3.23E-56 | Down |
| TRPM6 | -1.77309 | 1.22E-58 | 3.27E-56 | Down |
| DEFB1 | -2.55178 | 1.52E-58 | 4.00E-56 | Down |
| CMTM5 | -2.09171 | 5.45E-58 | 1.38E-55 | Down |
| EGFR | -1.54915 | 1.83E-57 | 4.52E-55 | Down |
| CNTNAP3B | -1.74757 | 2.05E-57 | 5.00E-55 | Down |
| RGMA | -1.60374 | 5.52E-57 | 1.33E-54 | Down |
| SLC6A2 | -2.44829 | 6.16E-57 | 1.47E-54 | Down |
| S100B | -2.13868 | 1.33E-56 | 3.13E-54 | Down |
| TCEAL5 | -2.01051 | 1.84E-56 | 4.26E-54 | Down |
| BNC1 | -2.02376 | 2.36E-56 | 5.42E-54 | Down |
| CAPN6 | -2.77238 | 2.42E-55 | 5.38E-53 | Down |
| MRAP2 | -1.82547 | 2.96E-55 | 6.49E-53 | Down |
| HS3ST4 | -2.39795 | 4.25E-55 | 9.22E-53 | Down |
| FERMT1 | -2.17779 | 5.31E-55 | 1.14E-52 | Down |
| ALX4 | -1.97764 | 5.57E-55 | 1.18E-52 | Down |
| IL33 | -1.94444 | 9.57E-55 | 2.01E-52 | Down |
| BLACAT1 | -2.49828 | 1.52E-54 | 3.15E-52 | Down |
| PTX3 | -2.07907 | 2.18E-54 | 4.43E-52 | Down |
| MMP7 | -2.31137 | 2.14E-54 | 4.39E-52 | Down |
| GRIA4 | -2.30409 | 7.77E-54 | 1.55E-51 | Down |
| CXCL2 | -1.78538 | 2.81E-53 | 5.49E-51 | Down |
| ANXA3 | -1.58592 | 3.06E-53 | 5.91E-51 | Down |
| BCL11A | -2.23126 | 1.21E-52 | 2.28E-50 | Down |
| LAMC2 | -1.76495 | 2.06E-52 | 3.79E-50 | Down |
| KRT16 | -3.21775 | 1.33E-51 | 2.40E-49 | Down |
| CALML3 | -1.99492 | 1.74E-51 | 3.08E-49 | Down |
| KCNJ16 | -2.20541 | 2.75E-51 | 4.84E-49 | Down |
| RERGL | -2.18043 | 7.27E-51 | 1.24E-48 | Down |
| CADM3 | -1.94715 | 1.13E-50 | 1.92E-48 | Down |
| SLC6A14 | -2.99008 | 2.68E-50 | 4.43E-48 | Down |
| LY6D | -2.93213 | 3.38E-50 | 5.54E-48 | Down |
| SAA2 | -2.09633 | 3.99E-50 | 6.48E-48 | Down |
| WIF1 | -2.98829 | 4.73E-50 | 7.56E-48 | Down |
| OPRPN | -3.09876 | 8.43E-50 | 1.34E-47 | Down |
| SLC7A3 | -2.1906 | 1.00E-49 | 1.57E-47 | Down |
| ADGRD2 | -2.08109 | 2.70E-49 | 4.11E-47 | Down |
| PTPRZ1 | -2.67539 | 4.12E-49 | 6.08E-47 | Down |
| KIT | -1.56811 | 5.35E-49 | 7.85E-47 | Down |
| PKP1 | -2.43287 | 6.88E-49 | 1.00E-46 | Down |
| SYN2 | -1.53905 | 1.02E-48 | 1.47E-46 | Down |
| LEFTY2 | -2.10546 | 1.15E-48 | 1.64E-46 | Down |
| KCNH8 | -1.97344 | 1.56E-48 | 2.19E-46 | Down |
| FAM107A | -1.59661 | 1.91E-48 | 2.66E-46 | Down |
| SLC34A2 | -3.1411 | 2.88E-48 | 3.98E-46 | Down |
| OLFM4 | -3.25074 | 1.68E-47 | 2.25E-45 | Down |
| CDH3 | -1.65158 | 2.83E-47 | 3.66E-45 | Down |
| ACTG2 | -2.1023 | 3.69E-47 | 4.67E-45 | Down |
| OCA2 | -2.33233 | 6.24E-47 | 7.85E-45 | Down |
| JCHAIN | -2.18604 | 7.53E-47 | 9.36E-45 | Down |
| ETV3L | -1.90594 | 1.75E-46 | 2.07E-44 | Down |
| LGR6 | -2.06003 | 2.06E-46 | 2.42E-44 | Down |
| KRT15 | -2.28754 | 2.50E-46 | 2.86E-44 | Down |
| ELN | -1.55745 | 2.40E-46 | 2.75E-44 | Down |
| PTN | -1.65544 | 3.17E-46 | 3.59E-44 | Down |
| DACT2 | -1.8989 | 4.10E-46 | 4.63E-44 | Down |
| MMP20 | -1.98901 | 5.18E-46 | 5.81E-44 | Down |
| NTF4 | -1.78524 | 6.83E-46 | 7.58E-44 | Down |
| CDHR1 | -1.59848 | 7.42E-46 | 8.18E-44 | Down |
| FDCSP | -3.51103 | 1.15E-45 | 1.26E-43 | Down |
| KRT6C | -2.48835 | 1.74E-45 | 1.87E-43 | Down |
| TGM5 | -2.01039 | 2.62E-45 | 2.78E-43 | Down |
| SLC27A6 | -2.0473 | 3.66E-45 | 3.84E-43 | Down |
| NPY2R | -2.32109 | 4.85E-45 | 5.01E-43 | Down |
| S100A2 | -2.13266 | 6.23E-45 | 6.37E-43 | Down |
| STAC | -1.55604 | 6.79E-45 | 6.83E-43 | Down |
| FOLR1 | -2.63734 | 6.91E-45 | 6.91E-43 | Down |
| PPP1R14C | -2.32413 | 1.01E-44 | 1.00E-42 | Down |
| CCL19 | -2.42272 | 1.83E-44 | 1.79E-42 | Down |
| MEOX1 | -1.69573 | 2.45E-44 | 2.38E-42 | Down |
| PI3 | -2.64012 | 1.18E-43 | 1.10E-41 | Down |
| TF | -2.00589 | 6.53E-43 | 5.85E-41 | Down |
| ALKAL2 | -1.83968 | 8.20E-43 | 7.30E-41 | Down |
| FABP7 | -3.18544 | 1.20E-42 | 1.06E-40 | Down |
| CHRDL1 | -2.02841 | 1.34E-42 | 1.18E-40 | Down |
| SCARA5 | -2.25207 | 2.29E-42 | 1.95E-40 | Down |
| FOXC1 | -1.60087 | 4.66E-42 | 3.87E-40 | Down |
| C6orf15 | -2.23507 | 1.10E-41 | 8.93E-40 | Down |
| PRSS12 | -1.82261 | 8.78E-41 | 6.96E-39 | Down |
| VGLL1 | -2.93607 | 1.74E-40 | 1.35E-38 | Down |
| KLK11 | -3.18751 | 2.87E-40 | 2.18E-38 | Down |
| LEMD1 | -2.24428 | 1.18E-39 | 8.69E-38 | Down |
| SPOCK3 | -1.70898 | 3.81E-39 | 2.72E-37 | Down |
| DCHS2 | -1.59553 | 5.54E-39 | 3.84E-37 | Down |
| C10orf90 | -1.7639 | 9.21E-39 | 6.30E-37 | Down |
| BPI | -1.90617 | 1.32E-38 | 8.97E-37 | Down |
| CCL21 | -2.36056 | 2.13E-38 | 1.42E-36 | Down |
| ANGPTL7 | -1.79996 | 1.04E-37 | 6.63E-36 | Down |
| MAPK4 | -2.30089 | 1.51E-37 | 9.49E-36 | Down |
| MARCHF4 | 1.562604 | 1.71E-37 | 1.06E-35 | Up |
| KRT6A | -2.88827 | 5.80E-37 | 3.48E-35 | Down |
| SLCO1A2 | -1.95342 | 1.46E-36 | 8.63E-35 | Down |
| IRX4 | -2.08393 | 3.06E-36 | 1.74E-34 | Down |
| CLEC10A | -1.52727 | 1.35E-35 | 7.21E-34 | Down |
| ITPRID1 | -2.08281 | 3.68E-35 | 1.90E-33 | Down |
| TCEAL2 | -1.70011 | 4.93E-35 | 2.52E-33 | Down |
| GDF10 | -1.55663 | 5.89E-35 | 2.99E-33 | Down |
| PIK3C2G | -1.88661 | 7.73E-35 | 3.90E-33 | Down |
| LGALS7B | -1.82356 | 9.80E-35 | 4.90E-33 | Down |
| TEX19 | 1.593833 | 9.92E-35 | 4.94E-33 | Up |
| KLK13 | -2.10821 | 1.68E-34 | 8.21E-33 | Down |
| WNT6 | -1.77046 | 2.74E-34 | 1.31E-32 | Down |
| SLPI | -1.96467 | 2.89E-34 | 1.38E-32 | Down |
| C1QL2 | -1.96531 | 6.07E-34 | 2.82E-32 | Down |
| RARRES1 | -1.53018 | 5.30E-34 | 2.47E-32 | Down |
| ALDH3A1 | -1.54888 | 1.99E-33 | 8.94E-32 | Down |
| ACKR1 | -2.14932 | 1.87E-33 | 8.46E-32 | Down |
| BRINP1 | -1.80191 | 2.34E-33 | 1.05E-31 | Down |
| PI16 | -2.17689 | 4.03E-33 | 1.73E-31 | Down |
| ASTN1 | -1.53271 | 4.43E-33 | 1.90E-31 | Down |
| VTCN1 | -1.96028 | 3.84E-33 | 1.66E-31 | Down |
| C4BPA | -1.88184 | 8.65E-33 | 3.61E-31 | Down |
| CHST9 | -1.83403 | 1.32E-32 | 5.43E-31 | Down |
| RGR | -1.72028 | 1.52E-32 | 6.24E-31 | Down |
| GJB3 | -1.95904 | 1.96E-32 | 7.99E-31 | Down |
| HPSE2 | -1.5349 | 2.35E-32 | 9.51E-31 | Down |
| C7 | -2.27431 | 2.60E-32 | 1.05E-30 | Down |
| TMEM132C | -2.06818 | 3.40E-32 | 1.35E-30 | Down |
| CA6 | -1.8812 | 4.30E-32 | 1.69E-30 | Down |
| CDH19 | -2.28902 | 2.47E-31 | 9.15E-30 | Down |
| PROM1 | -2.29264 | 4.14E-31 | 1.50E-29 | Down |
| LRFN2 | 1.572179 | 5.69E-31 | 2.04E-29 | Up |
| GPRIN2 | -1.82275 | 6.38E-31 | 2.28E-29 | Down |
| CHI3L2 | -1.6758 | 1.06E-30 | 3.73E-29 | Down |
| MUC15 | -2.36968 | 1.89E-30 | 6.51E-29 | Down |
| TUNAR | -1.91505 | 5.66E-30 | 1.87E-28 | Down |
| CD300LG | -1.85232 | 7.76E-30 | 2.55E-28 | Down |
| KIAA0319 | 1.628807 | 2.04E-29 | 6.43E-28 | Up |
| KRT9 | -1.62653 | 3.33E-29 | 1.03E-27 | Down |
| CXCL1 | -1.6193 | 9.15E-29 | 2.72E-27 | Down |
| GNG13 | 1.664134 | 1.12E-28 | 3.30E-27 | Up |
| HRCT1 | -1.50636 | 1.75E-28 | 5.10E-27 | Down |
| GJB6 | -1.87588 | 2.02E-28 | 5.87E-27 | Down |
| LTF | -2.25796 | 2.15E-28 | 6.24E-27 | Down |
| ROPN1B | -1.86107 | 5.27E-28 | 1.47E-26 | Down |
| RIC3 | -1.52543 | 6.98E-28 | 1.92E-26 | Down |
| KRT81 | -2.34878 | 3.12E-27 | 7.87E-26 | Down |
| ATP13A5 | -1.94228 | 4.02E-27 | 1.01E-25 | Down |
| SLC19A3 | -1.61891 | 1.18E-26 | 2.80E-25 | Down |
| MGAM2 | -1.76073 | 2.20E-26 | 5.07E-25 | Down |
| SHC4 | -1.52834 | 2.14E-26 | 4.94E-25 | Down |
| PLP1 | -1.54775 | 2.34E-26 | 5.36E-25 | Down |
| GSTA1 | -1.97422 | 2.62E-26 | 5.96E-25 | Down |
| PCOLCE2 | -1.55176 | 3.47E-26 | 7.79E-25 | Down |
| SPIB | -1.52432 | 2.03E-25 | 4.28E-24 | Down |
| CXCL6 | -1.53065 | 3.46E-25 | 7.12E-24 | Down |
| UGT8 | -1.62337 | 9.23E-25 | 1.80E-23 | Down |
| ERICH5 | -1.58139 | 1.04E-24 | 2.02E-23 | Down |
| SLC5A1 | -1.84552 | 1.57E-24 | 2.99E-23 | Down |
| PTCHD1 | -1.67351 | 2.33E-24 | 4.38E-23 | Down |
| MUC7 | -1.76803 | 2.76E-24 | 5.12E-23 | Down |
| ELF5 | -2.14308 | 7.32E-24 | 1.32E-22 | Down |
| PIGR | -2.09316 | 1.10E-23 | 1.93E-22 | Down |
| SBK2 | 1.656232 | 1.90E-23 | 3.25E-22 | Up |
| RHCG | -1.71547 | 2.22E-23 | 3.77E-22 | Down |
| MYT1 | 1.694201 | 2.29E-23 | 3.88E-22 | Up |
| CCN6 | -1.5007 | 2.38E-23 | 4.03E-22 | Down |
| TMPRSS3 | -1.51702 | 2.70E-23 | 4.54E-22 | Down |
| LCN2 | -1.8182 | 7.46E-23 | 1.20E-21 | Down |
| SLC6A15 | -1.89598 | 8.62E-23 | 1.38E-21 | Down |
| CIDEA | -1.90379 | 8.98E-23 | 1.44E-21 | Down |
| PRRT1B | 1.545135 | 3.44E-22 | 5.19E-21 | Up |
| MYL7 | -1.6257 | 5.01E-22 | 7.46E-21 | Down |
| FOXA1 | 1.571809 | 1.06E-21 | 1.53E-20 | Up |
| KRT23 | -1.52679 | 2.62E-21 | 3.63E-20 | Down |
| TTC6 | 1.527271 | 2.92E-21 | 4.03E-20 | Up |
| CXCL5 | -1.5203 | 6.87E-21 | 9.16E-20 | Down |
| CIDEC | -1.92819 | 1.22E-20 | 1.59E-19 | Down |
| NKX1-2 | -1.79003 | 1.90E-20 | 2.44E-19 | Down |
| ADH1B | -2.35879 | 2.38E-20 | 3.02E-19 | Down |
| OBP2B | -2.14482 | 3.17E-20 | 3.97E-19 | Down |
| TCL1A | -1.54941 | 3.99E-20 | 4.94E-19 | Down |
| MYBPC1 | -2.14837 | 3.89E-20 | 4.83E-19 | Down |
| SMR3B | -1.54565 | 6.38E-20 | 7.74E-19 | Down |
| DIO1 | 1.655559 | 4.76E-19 | 5.29E-18 | Up |
| CWH43 | -1.66336 | 8.79E-19 | 9.42E-18 | Down |
| LEP | -1.77638 | 1.10E-18 | 1.17E-17 | Down |
| ABCA13 | -1.56365 | 1.27E-18 | 1.34E-17 | Down |
| ADGRF1 | -1.51222 | 1.42E-18 | 1.48E-17 | Down |
| TCN1 | -1.97164 | 1.49E-18 | 1.55E-17 | Down |
| SCEL | -1.59756 | 3.75E-18 | 3.76E-17 | Down |
| ARHGAP40 | -1.9852 | 3.98E-18 | 3.96E-17 | Down |
| MS4A1 | -1.52482 | 6.76E-18 | 6.61E-17 | Down |
| NKAIN1 | 1.508048 | 4.79E-18 | 4.75E-17 | Up |
| CR2 | -1.62993 | 2.81E-17 | 2.58E-16 | Down |
| CGA | 2.054624 | 8.87E-17 | 7.69E-16 | Up |
| CLCA2 | -1.81152 | 9.32E-17 | 8.06E-16 | Down |
| TRARG1 | -1.92129 | 1.22E-16 | 1.04E-15 | Down |
| FABP4 | -1.62428 | 1.56E-16 | 1.32E-15 | Down |
| KCNJ3 | 2.406549 | 2.79E-16 | 2.30E-15 | Up |
| PPP1R1B | -1.51357 | 1.98E-16 | 1.66E-15 | Down |
| CPLX2 | 1.572958 | 5.36E-16 | 4.31E-15 | Up |
| DCX | -1.55295 | 6.67E-16 | 5.31E-15 | Down |
| CEACAM5 | 1.859148 | 7.65E-15 | 5.42E-14 | Up |
| ADIPOQ | -1.97617 | 2.04E-14 | 1.38E-13 | Down |
| ASCL1 | 1.797799 | 7.46E-14 | 4.72E-13 | Up |
| AQP5 | -1.5251 | 7.67E-13 | 4.34E-12 | Down |
| GPR139 | 1.568411 | 3.48E-12 | 1.83E-11 | Up |
| CRABP1 | -1.54151 | 1.69E-11 | 8.25E-11 | Down |
| TFAP2B | -1.50033 | 1.12E-08 | 3.96E-08 | Down |
| S100A7 | -1.56848 | 1.00E-07 | 3.18E-07 | Down |

**Supplementary table 4 | Univariate Cox regression identifying 50** **OS-associated DEGs**

| Gene | HR |  | HR.95L | HR.95H | *P*.value |
| --- | --- | --- | --- | --- | --- |
| ALDH3A1 | 0.669183 |  | 0.476851 | 0.939089 | 0.020154 |
| ALKAL2 | 0.789844 |  | 0.640662 | 0.973763 | 0.027182 |
| ALX4 | 0.842667 |  | 0.716988 | 0.990377 | 0.037774 |
| ASCL1 | 1.087909 |  | 1.003373 | 1.179568 | 0.041197 |
| CCL19 | 0.890541 |  | 0.834053 | 0.950855 | 0.000526 |
| CDH19 | 0.769506 |  | 0.615938 | 0.961362 | 0.02106 |
| CEACAM5 | 1.063108 |  | 1.001974 | 1.127972 | 0.042844 |
| CHI3L1 | 0.913468 |  | 0.848841 | 0.983016 | 0.015627 |
| CLCA4 | 0.638619 |  | 0.440533 | 0.925774 | 0.01793 |
| CLEC10A | 0.861321 |  | 0.76448 | 0.970429 | 0.014158 |
| COL17A1 | 0.904209 |  | 0.831899 | 0.982803 | 0.017891 |
| CPLX2 | 1.112892 |  | 1.008737 | 1.227802 | 0.032886 |
| CWH43 | 1.140129 |  | 1.008473 | 1.288971 | 0.036194 |
| CXCL1 | 0.831454 |  | 0.728123 | 0.949449 | 0.006409 |
| CXCL2 | 0.863059 |  | 0.764494 | 0.974331 | 0.017301 |
| ECRG4 | 0.881181 |  | 0.793481 | 0.978574 | 0.018035 |
| FABP7 | 0.89916 |  | 0.824816 | 0.980204 | 0.015776 |
| FDCSP | 0.940733 |  | 0.891216 | 0.993002 | 0.026793 |
| FGFBP1 | 0.847746 |  | 0.740008 | 0.971169 | 0.017227 |
| IL33 | 0.871995 |  | 0.787286 | 0.965819 | 0.008614 |
| JCHAIN | 0.884566 |  | 0.831428 | 0.9411 | 0.000104 |
| KIAA0319 | 1.248199 |  | 1.04147 | 1.495963 | 0.016404 |
| KRT14 | 0.932849 |  | 0.882863 | 0.985666 | 0.013369 |
| KRT15 | 0.912356 |  | 0.850294 | 0.978948 | 0.010713 |
| KRT17 | 0.92131 |  | 0.868879 | 0.976905 | 0.006114 |
| KRT5 | 0.924607 |  | 0.874015 | 0.978127 | 0.006328 |
| LAMB3 | 0.879638 |  | 0.796609 | 0.971321 | 0.011238 |
| LAMC2 | 0.910164 |  | 0.830066 | 0.997992 | 0.045205 |
| LTF | 0.942961 |  | 0.896851 | 0.991441 | 0.021675 |
| MEOX1 | 0.863793 |  | 0.768413 | 0.971011 | 0.014177 |
| MMP7 | 0.921747 |  | 0.858511 | 0.98964 | 0.02463 |
| MRAP2 | 0.813061 |  | 0.692135 | 0.955116 | 0.011772 |
| MS4A1 | 0.893952 |  | 0.80138 | 0.997216 | 0.044437 |
| OSR1 | 0.861505 |  | 0.762149 | 0.973814 | 0.017108 |
| PIGR | 0.883769 |  | 0.819117 | 0.953523 | 0.001433 |
| RARRES1 | 0.912753 |  | 0.835448 | 0.997211 | 0.043196 |
| RNF186 | 0.551518 |  | 0.332337 | 0.915253 | 0.0213 |
| S100B | 0.866182 |  | 0.785731 | 0.95487 | 0.003871 |
| SAA1 | 0.919491 |  | 0.857246 | 0.986255 | 0.018927 |
| SAA2 | 0.880386 |  | 0.78129 | 0.992051 | 0.036533 |
| SFRP1 | 0.930946 |  | 0.873839 | 0.991786 | 0.026737 |
| SLC27A6 | 0.866056 |  | 0.758793 | 0.988481 | 0.033031 |
| SLC7A3 | 0.775795 |  | 0.623521 | 0.965256 | 0.022777 |
| SPIB | 0.843394 |  | 0.738033 | 0.963795 | 0.012364 |
| STAC2 | 0.932394 |  | 0.874816 | 0.993762 | 0.031369 |
| TACR1 | 0.841566 |  | 0.717643 | 0.986888 | 0.033807 |
| TCN1 | 0.891733 |  | 0.82773 | 0.960685 | 0.002566 |
| TEX19 | 1.313404 |  | 1.101508 | 1.566063 | 0.00239 |
| TP63 | 0.864983 |  | 0.773572 | 0.967195 | 0.010919 |
| TSLP | 0.536548 |  | 0.351833 | 0.818239 | 0.003832 |
