# Supplementary figures and images for: Integrating bulk and single-cell RNA sequencing analysis to reveal characterization of mechanical stimulus-related genes and prognostic signatures in breast cancer
Source: Breast Cancer Res. 2025 Nov 13;27:204. doi: 10.1186/s13058-025-02130-6 (PMC12616973; doi:10.1186/s13058-025-02130-6)

Fig. 8A

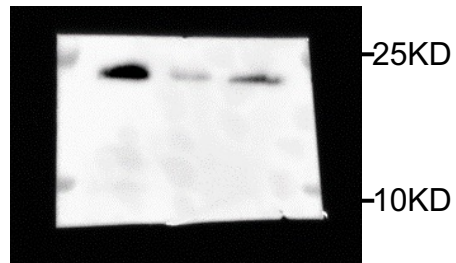

TEX19

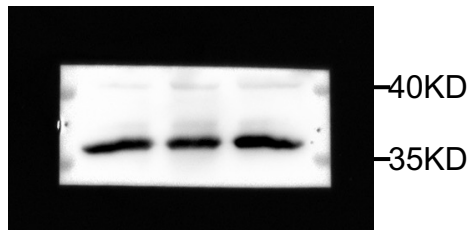

GAPDH

Fig. 8B

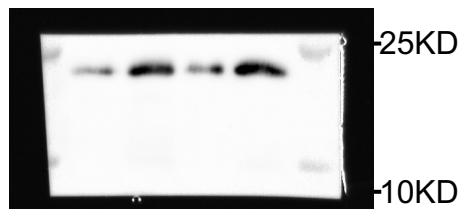

TEX19

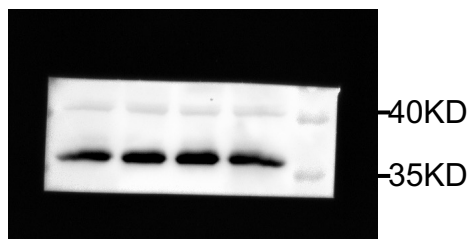

GAPDH

Supplement: Supplementary file 3 — Supplementary file3. [file 13058_2025_2130_MOESM3_ESM.pdf]
